# Cocultivation of Anaerobic Fungi with Rumen Bacteria Establishes an Antagonistic Relationship

**DOI:** 10.1128/mBio.01442-21

**Published:** 2021-08-17

**Authors:** Candice L. Swift, Katherine B. Louie, Benjamin P. Bowen, Casey A. Hooker, Kevin V. Solomon, Vasanth Singan, Chris Daum, Christa P. Pennacchio, Kerrie Barry, Vaithiyalingam Shutthanandan, James E. Evans, Igor V. Grigoriev, Trent R. Northen, Michelle A. O’Malley

**Affiliations:** a Department of Chemical Engineering, University of California Santa Barbara, Santa Barbara, California, USA; b U.S. Department of Energy Joint Genome Institute, Lawrence Berkeley National Laboratory, Berkeley, California, USA; c Department of Agricultural & Biological Engineering, Purdue University, West Lafayette, Indiana, USA; d Environmental Molecular Sciences Laboratory, Pacific Northwest National Laboratory, Richland, Washington, USA; e Environmental Genomics and Systems Biology Division, Lawrence Berkeley National Laboratory, Berkeley, California, USA; f Department of Plant and Microbial Biology, University of California Berkeley, Berkeley, California, USA; g Joint BioEnergy Institute (JBEI), Emeryville, California, USA; University of California, Berkeley

**Keywords:** RNA-seq, transcriptomics, cocultivation, secondary metabolism, fungi, anaerobe, anaerobic fungi

## Abstract

Anaerobic gut fungi (Neocallimastigomycetes) live in the digestive tract of large herbivores, where they are vastly outnumbered by bacteria. It has been suggested that anaerobic fungi challenge growth of bacteria owing to the wealth of biosynthetic genes in fungal genomes, although this relationship has not been experimentally tested. Here, we cocultivated the rumen bacteria *Fibrobacter succinogenes* strain UWB7 with the anaerobic gut fungi *Anaeromyces robustus* or *Caecomyces churrovis* on a range of carbon substrates and quantified the bacterial and fungal transcriptomic response. Synthetic cocultures were established for at least 24 h, as verified by active fungal and bacterial transcription. *A. robustus* upregulated components of its secondary metabolism in the presence of *Fibrobacter succinogenes* strain UWB7, including six nonribosomal peptide synthetases, one polyketide synthase-like enzyme, and five polyketide synthesis O-type methyltransferases. Both *A. robustus* and *C. churrovis* cocultures upregulated *S*-adenosyl-l-methionine (SAM)-dependent methyltransferases, histone methyltransferases, and an acetyltransferase. Fungal histone 3 lysine 27 trimethylation marks were more abundant in coculture, and heterochromatin protein-1 was downregulated. Together, these findings suggest that fungal chromatin remodeling occurs when bacteria are present. *F. succinogenes* strain UWB7 upregulated four genes in coculture encoding drug efflux pumps, which likely protect the cell against toxins. Furthermore, untargeted nonpolar metabolomics data revealed at least one novel fungal metabolite enriched in coculture, which may be a defense compound. Taken together, these data suggest that *A. robustus* and *C. churrovis* produce antimicrobials when exposed to rumen bacteria and, more broadly, that anaerobic gut fungi are a source of novel antibiotics.

## INTRODUCTION

Microbial antagonism can take many forms: antibiosis (the production by an organism of a compound that inhibits or kills another organism), competition for nutrients and space, parasitism, and others (1). Although often discussed in the context of biological control agents that protect postharvest crops (1–3), microbial antagonism has also been recognized to have a profound impact on microbial communities, especially host-associated communities (4). For example, microbial antagonism can increase microbial diversity (5, 6), protect against invasion by pathogens (7), and drive genome evolution through the acquisition of genetic material from killed cells (8). Mathematical modeling suggests that communities dominated by antagonistic relationships are more stable and resilient to perturbations than those dominated by cooperative relationships (9).

Microbial relationships, especially antagonistic ones, within the rumen microbiome are complex and not well-characterized. In particular, knowledge of rumen fungi (class Neocallimastigomycetes) and their interactions with other microbial community members is lacking. Rumen fungi, also referred to as anaerobic gut fungi, thrive in the digestive tracts of large herbivores as part of a biomass-degrading consortium with bacteria, methanogenic archaea, and protozoa (10, 11). Bacteria outnumber fungi in the rumen by at least 4 orders of magnitude (10, 11). Cocultivation of fungi with bacteria suggests that the nature of the interaction between rumen fungi and bacteria depends on the specific fungal-bacterial pairing. Antagonistic relationships, in which the cellulolytic activity of the fungus was inhibited, were observed between *Ruminococcus flavefaciens* and *Neocallimastix frontalis* MCH3 or *Piromyces communis* FL (12), *Piromyces communis*, and Selenomonas ruminantium (13), as well as *R. flavefaciens* and *Orpinomyces joyonii* or *N. frontaslis* (14). Some previous studies of rumen fungi cocultivated with the cellulolytic rumen bacteria Fibrobacter succinogenes have shown no effect on biomass degradation (12, 14, 15), implying neither mutualism nor antagonism between these organisms. However, Joblin and colleagues found that *F. succinogenes* inhibited the degradation of ryegrass stems by *N. frontalis* in coculture with Methanobrevibacter smithii, whereas the presence of *F. succinogenes* enhanced degradation by cocultures of *Caecomyces* spp. with M. smithii (16). In a separate study by Roger and colleagues (14), the presence of *F. succinogenes* had no impact on the degradation of wheat straw or maize stem by *N. frontalis* or *Orpinomyces* (*Neocallimastix*) *joyonii*.

Coculture transcriptomics has proven to be a valuable tool by which to investigate the nature of microbial interactions, as demonstrated in recent publications (17–21). For example, RNA sequencing (RNA-seq) of the anaerobic fungus *Anaeromyces robustus* in coculture with the methanogen Methanobacterium bryantii revealed that the fungus upregulated 105 genes encoding carbohydrate-active enzymes (CAZymes), representing 12% of total predicted CAZymes (18). Coculture transcriptomics and fermentation profiling of *Pecoramyces* sp. strain *F1* with *Methanobrevibacter thaueri* also supported a syntrophic fungal-methanogen relationship (21). However, RNA sequencing of both fungi and bacteria in coculture remains difficult due to the technical challenge of depleting rRNA from both microbes. Here, we cocultivated pairings of rumen fungi with *Fibrobacter succinogenes* strain UWB7 (2), and performed the first dual transcriptomic characterization of a rumen bacterium and fungus in coculture. By this approach, we tested the hypothesis that the relationship between *F. succinogenes* strain UWB7 and anaerobic gut fungi is antagonistic. Furthermore, we performed untargeted nonpolar metabolomics to investigate whether the introduction of *F. succinogenes* strain UWB7 triggers the production of possible defense compounds by the anaerobic fungus. Specifically, we cultured *Anaeromyces robustus* with *F. succinogenes* strain UWB7 on crystalline cellulose (Avicel PH-101; Sigma) or switchgrass as well as *Caecomyces churrovis* with *F. succinogenes* strain UWB7 on switchgrass (see Fig. S1 in the supplemental material) and compared them to the respective fungal and bacterial monocultures.

10.1128/mBio.01442-21.6 FIG S1 Experimental design of fungal-bacterial cocultures. (A) Schematic of the cocultivation pairings of anaerobic fungi with the rumen bacteria *F. succinogenes* strain UWB7 on two different carbon substrates. Fungal cultures were grown for 24 h prior to the introduction of *F. succinogenes* strain UWB7 and subsequently cocultured for the duration listed below each Venn diagram. (B) Detailed experimental schematic of the preparation of cultures for transcriptomics and metabolomics. Seed cultures of fungus and *F. succinogenes* strain UWB7 were inoculated into serum bottles and grown for 4 days (fungus) or 24 h (bacteria). These cultures were used as the inoculum for Hungate tubes (4 biological replicates per condition), which were subsequently harvested after at least 48 h of growth post inoculation. For cocultures, the fungus was grown in isolation for 24 h prior to the introduction of bacteria. Download FIG S1, DOCX file, 0.2 MB. Copyright © 2021 Swift et al. 2021 Swift et al. https://creativecommons.org/licenses/by/4.0/ This content is distributed under the terms of the Creative Commons Attribution 4.0 International license.

## RESULTS AND DISCUSSION

Secondary metabolites, although not strictly necessary for the growth or survival of an organism under all growth conditions (22), are often secreted during antagonistic relationships between microorganisms (23–25). Previous work mining the high-quality genomes of anaerobic fungi revealed that anaerobic fungi are capable of synthesizing secondary metabolites (26). *A. robustus* and *C. churrovis* encode 43 and 32 biosynthetic enzymes, respectively, for various classes of secondary metabolites, including nonribosomal peptide synthetases (NRPSs) and polyketide synthases (PKSs) (26). We hypothesized that some of the secondary metabolites produced by *A. robustus* and *C. churrovis* are compounds used for regulation, defense, or competition against rumen bacteria.

### Cocultivation with rumen bacteria induces stress in anaerobic fungi and activates components of fungal secondary metabolism.

**FIG 1:**
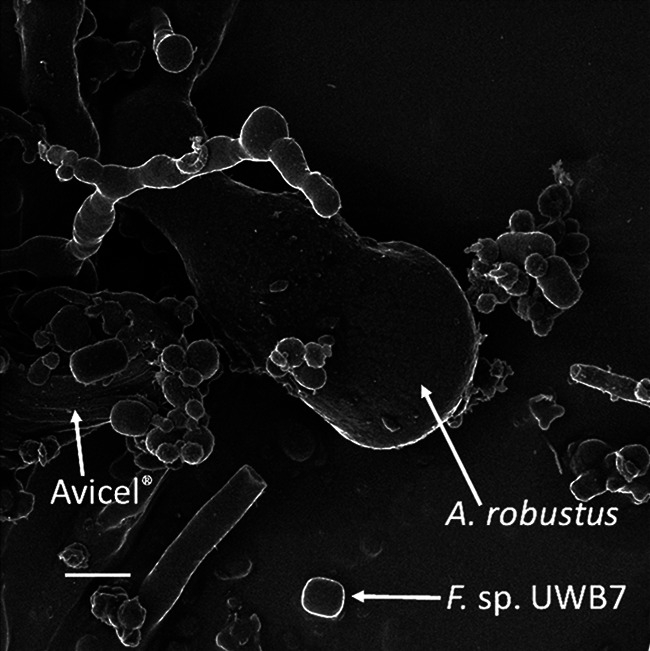
Helium ion micrograph of *A. robustus* grown in coculture with *Fibrobacter succinogenes* strain UWB7 on Avicel. The presence of *A. robustus* is indicated by a sporangium, and the presence of *Fibrobacter succinogenes* strain UWB7 is indicated by single cells. The scale bar in the lower left corner represents 2.00 μm. Image brightness was adjusted for clarity.

**FIG 2:**
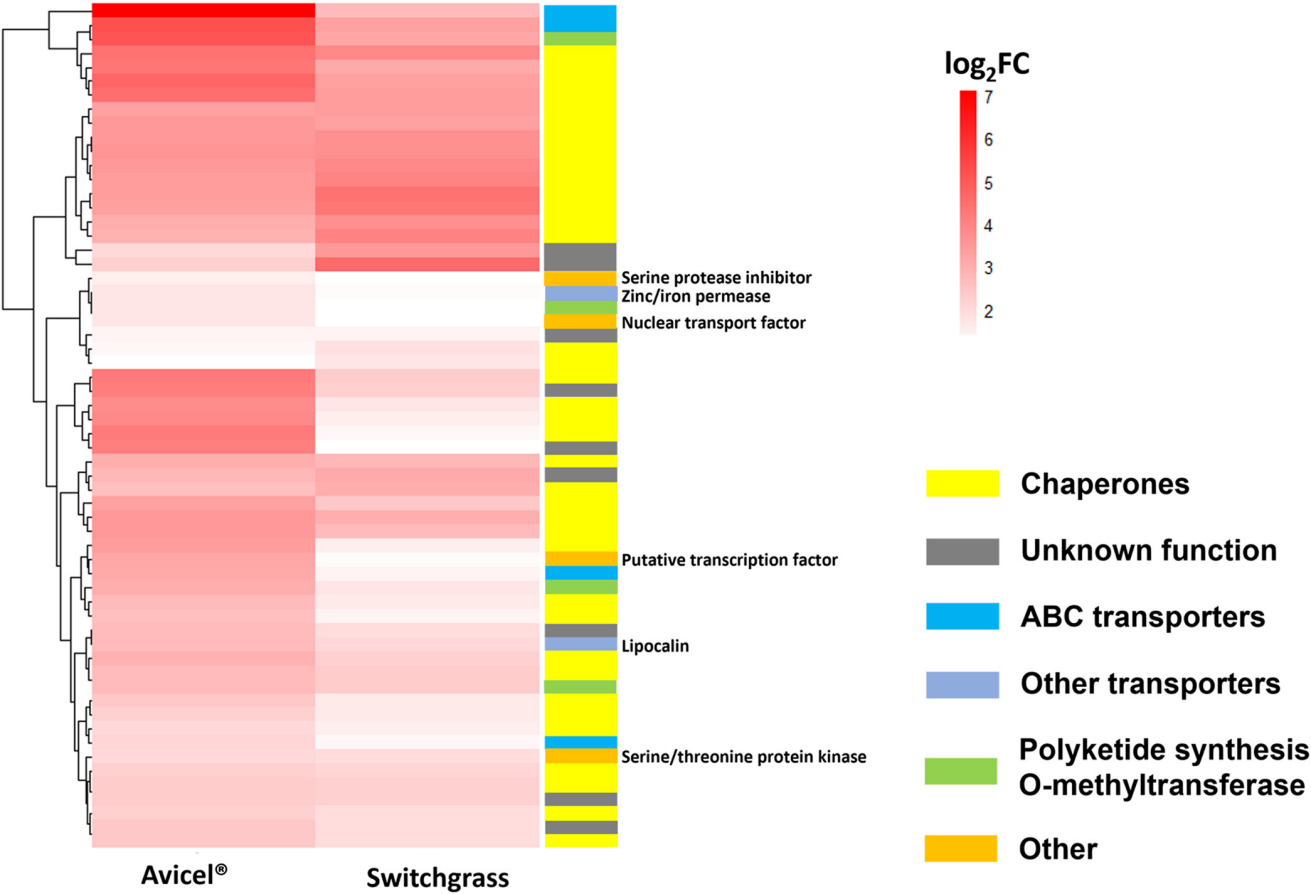
*A. robustus* upregulates transporters, chaperones, and O-methyltransferases in response to cocultivation with *F. succinogenes* strain UWB7 on different carbon substrates. Heatmap represents the log_2_ fold change of *A. robustus* transcript abundance in coculture with *F. succinogenes* strain UWB7 relative to respective fungal monoculture at mid-log phase on two different carbon substrates (Avicel or switchgrass). Only fungal transcripts upregulated at least 2-fold with an adjusted *P* value of <0.05 in coculture versus monoculture are shown. Putative functions are designated as assigned in MycoCosm (30), KOG (48), or InterPro (81).

Although not stable for many generations of batch passaging, anaerobic fungi can grow with *F. succinogenes* strain UWB7 for a sufficient duration to capture transcriptional responses from both organisms in coculture. *A. robustus* or *C. churrovis* was grown in isolation for 24 h prior to the introduction of *F. succinogenes* strain UWB7 (see Fig. S1 in the supplemental material). Fungi and bacteria were cocultured until the fungus reached mid-log phase, at which point the cocultures and corresponding fungal monocultures were harvested for RNA extraction and sequencing. In response to the presence of *F. succinogenes* strain UWB7 (Fig. 1), cultures of *A. robustus* or *C. churrovis* both upregulated genes encoding stress response proteins (chaperones), indicating that the presence of the bacteria invoked a fungal stress response (Fig. 2; see also Data Sets S1 to S3, available at https://github.com/cswift3/anaerobic_fungi_Fibrobacter_co-culture). Fungal stress was observed during growth on switchgrass or Avicel PH-101. Gene set enrichment analysis (27) supported enrichment of small heat shock proteins upregulated in coculture (Data Sets S4 to S6 at the URL mentioned above). However, roughly 10 times more genes were differentially regulated comparing fungal coculture to monoculture during growth on Avicel relative to growth on switchgrass (Table S2 and Data Sets S1 to S3 at the URL mentioned above). This difference may reflect the preference of *F. succinogenes* strain UWB7, a cellulose-degrading specialist (28), for crystalline cellulose over complex plant matter, resulting in both more robust bacterial growth and a stronger bacterial signal to the fungi.

**FIG 3:**
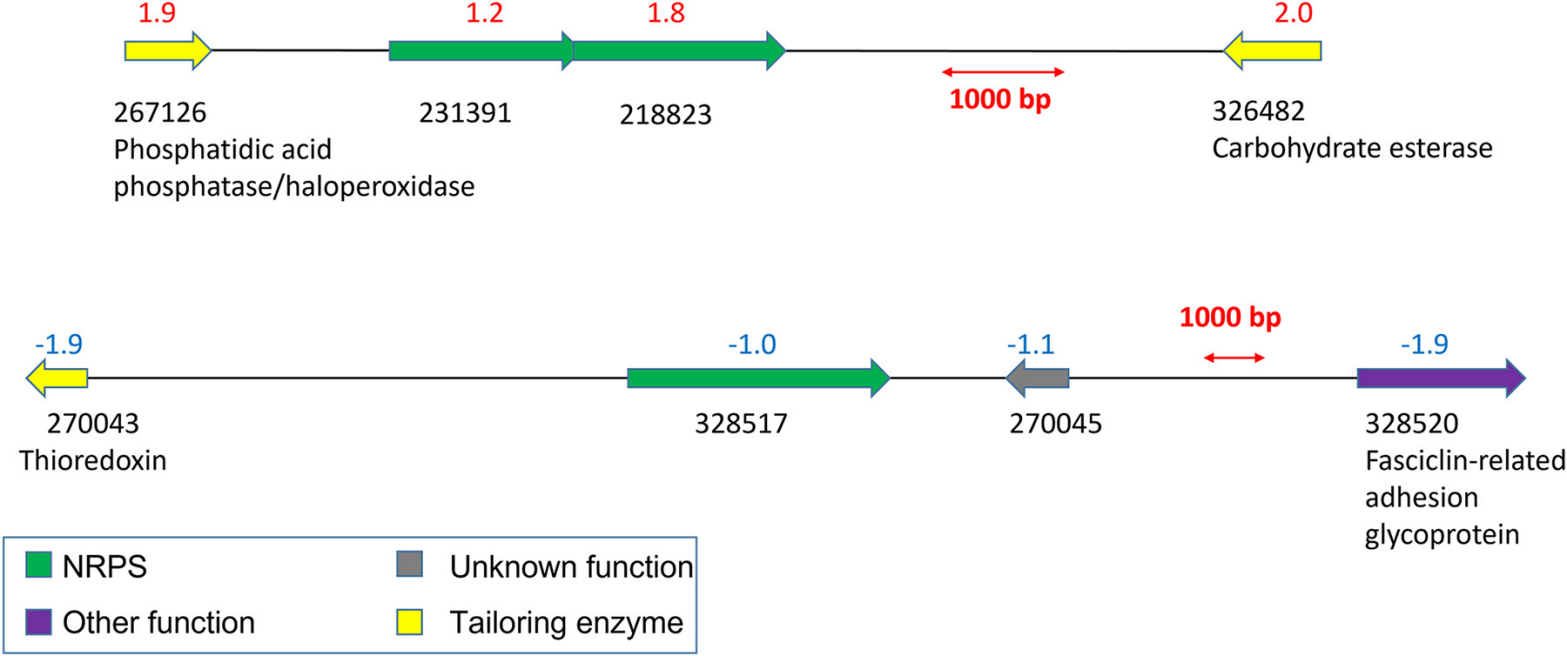
Genes that were coordinately regulated in coculture of *A. robustus* with *Fibrobacter* sp. strain UWB7 versus fungal monocultures. All cultures were grown on Avicel. Log_2_ fold change is shown above each gene, and the MycoCosm protein ID is shown below each gene.

In addition to a general stress response, *A. robustus* upregulated six nonribosomal peptide synthetases (NRPSs) and one polyketide synthase (PKS)-like enzyme (Table 1) at least 2-fold (adjusted *P* value of <0.05) in coculture with *F. succinogenes* strain UWB7 compared to *A. robustus* monoculture (both cultures grown on Avicel). These genes and others related to fungal secondary metabolism were previously annotated (26). Notably, these genes do not have homologs in *C. churrovis*, as determined by a bidirectional protein BLAST (29) in the MycoCosm portal (30). This suggests that the aspects of the response to *F. succinogenes* strain UWB7 related to secondary metabolism are specific to the fungal strain. Neighboring genes were coregulated with a pair of NRPS genes (Fig. 3). One NRPS gene cluster was downregulated in the Avicel coculture (Fig. 3). In higher-order fungi, secondary metabolites are linked to different developmental stages of fungi (31–33), and some of the secondary metabolites from anaerobic fungi may also serve such a purpose. In addition, five genes encoding putative polyketide O-methyltransferases were upregulated with a log_2_ fold change of 2.7 or greater (Table S3). Surprisingly, a predicted protein (271870) containing a condensation domain, which would normally form a modular domain on an NRPS (34), was upregulated 2-fold in coculture relative to monoculture. Taken together, these data suggest that *A. robustus* regulates polyketide and nonribosomal peptide synthesis in response to microbial challenge by *F. succinogenes* strain UWB7.

**TABLE 1:** Differentially regulated biosynthetic genes for secondary metabolites^*a*^

MycoCosm protein ID	SM type	Log_2_ fold change	Scaffold
*A. robustus* (Avicel)			
193122	NRPS	2.9	480
294553	NRPS	2.9	182
271076	NRPS	2	279
231391*	NRPS	1.8	77
266215	PKS-like	1.6	49
218823*	NRPS	1.2	77
330657	NRPS	1.1	540
328517*	PKS	−1.0	207
*C. churrovis* (switchgrass)			
17094	PKS	−3.6	116

a Genes marked with an asterisk in the protein ID column indicate the gene is coregulated with neighboring genes (Fig. 2). Adjusted *P*  value of <0.05. The log_2_ fold change refers to the fungus in coculture with *Fibrobacter* compared to the fungus grown in monoculture.

10.1128/mBio.01442-21.4 TABLE S3 Anaerobic gut fungi *A. robustus* and *C. churrovis* upregulate genes with potential functions in secondary metabolism or chromatin remodeling when cocultured with *F. succinogenes* strain UWB7. Genes with putative functions in secondary metabolism include those involved in polyketide and nonribosomal peptide synthesis as well as SAM-dependent methyltransferases that are potentially involved in the regulation of secondary metabolism. Adjusted *P* value was less than 0.05 for all comparisons. Download Table S3, DOCX file, 0.02 MB. Copyright © 2021 Swift et al. 2021 Swift et al. https://creativecommons.org/licenses/by/4.0/ This content is distributed under the terms of the Creative Commons Attribution 4.0 International license.

### Anaerobic fungi regulate their secondary metabolism via epigenetic modifications in the presence of rumen bacteria.

LaeA is reported to modulate gene expression of biosynthetic gene clusters (BGCs) via chromatin remodeling (35), and studies have found that epigenetic modifications such as histone acetylation or methylation can regulate expression of biosynthetic gene clusters in fungi (35–37). Both *A. robustus* and *C. churrovis* upregulated genes encoding *S*-adenosyl methionine (SAM)-dependent methyltransferases when cocultured with *F. succinogenes* strain UWB7 (Table S3). One of these proteins may perform a function similar to that of LaeA or Lae1, which act in concert with other proteins, including proteins containing velvet domains (38), as global regulators of secondary metabolism in higher-order fungi (39–41). In previous work, we identified a homolog of the velvet-containing gene *vosA* (42, 43) in *C. churrovis* (26). We hypothesize that anaerobic gut fungi remodel chromatin via histone modifications to modulate their secondary metabolism, similar to what has been suggested for higher-order fungi (35, 44). When cocultured with *F. succinogenes* strain UWB7, both *C. churrovis* and *A. robustus* upregulated genes with putative functions in histone methylation or acetylation (Table S3), which are both modifications known to be involved in determining heterochromatin or euchromatin locations (45). It is possible that one of the highly upregulated methyltransferases in coculture acts as a global regulator of secondary metabolism in anaerobic gut fungi, similar to LaeA and Lae1. However, the distant evolutionary relationship between Neocallimastigomycetes and higher-order fungi as well as the current lack of genetic tractability of rumen fungi makes previous approaches used to pinpoint LaeA homologs unreliable at this time.

To test whether there were differences in the amount of histone 3-lysine 4 and histone 3-lysine 27 trimethylation marks (H3K4me3 and H3K27me3, respectively) between fungal monocultures and fungal cocultures with *Fibrobacter succinogenes* strain UWB7, we performed Western blotting on monoculture and coculture cell lysates using antibodies raised to S. cerevisiae H3K4me3 and H3K27me3 (Fig. S2). The exposure time for the H3 loading control increased by a factor of 10 between monoculture and coculture, indicating decreased fungal biomass in coculture. However, the exposure times were nearly equivalent between H3K27me3 blots of monocultures and cocultures, indicating that despite the decreased fungal biomass in coculture, there was an increased proportion of H3K27me3 marks. H3K27me3 is known to be a repressor of transcription, whereas H3K4me3 is an activating mark (46). Consistent with the downregulation of genes due to the enhancement of H3K27me3 marks in coculture, more genes were downregulated than upregulated when comparing *A. robustus* cocultures to monocultures during growth on Avicel (Table S2). These results support that epigenetic modifications influence gene regulation when *A. robustus* is exposed to *F. succinogenes* strain UWB7. Furthermore, two genes encoding heterochromatin-associated protein HP1 were greater than 2-fold downregulated in coculture (protein identifiers [IDs] 290815 and 266437), and another gene (280338) encoding a homolog of the WSTF-ISWI chromatin remodeling complex, which has been implicated in the replication of heterochromatin (47), was 8-fold downregulated in coculture (Data Set S1 at https://github.com/cswift3/anaerobic_fungi_Fibrobacter_co-culture). In aspergilli, heterochromatin protein-1 and H3K9me3 marks have been associated with the repression of secondary metabolite gene clusters (35, 44). Taken together, these findings suggest that the secondary metabolism of anaerobic fungi is regulated via epigenetic marks and chromatin remodeling, consistent with higher-order fungi.

10.1128/mBio.01442-21.2 TABLE S1 Assessment of the effectiveness of ribosomal depletion in sequenced libraries of cocultures of *C. churrovis* and *F. succinogenes* strain UWB7 and monocultures of *F. succinogenes* strain UWB7 grown on switchgrass. Reads were categorized by SortMeRNA (72) with reference to the SILVA (86) and Rfam (87) databases. Read percentages are based on quality-filtered reads as described in the text. Download Table S1, DOCX file, 0.02 MB. Copyright © 2021 Swift et al. 2021 Swift et al. https://creativecommons.org/licenses/by/4.0/ This content is distributed under the terms of the Creative Commons Attribution 4.0 International license.

10.1128/mBio.01442-21.3 TABLE S2 Number of differentially regulated fungal genes in coculture with *F. succinogenes* strain UWB7 compared to fungal monoculture (absolute log_2_ fold change of >1 and adjusted *P* value of <0.05). AV, Avicel; SG, switchgrass. Download Table S2, DOCX file, 0.01 MB. Copyright © 2021 Swift et al. 2021 Swift et al. https://creativecommons.org/licenses/by/4.0/ This content is distributed under the terms of the Creative Commons Attribution 4.0 International license.

10.1128/mBio.01442-21.7 FIG S2 (A) Western blot images of *A. robustus* monocultures grown on Avicel with rabbit antibodies against histone 3 (H3), trimethylated lysine 4 on histone 3 (H3K4me3), and trimethylated lysine 27 on histone 3 (H3K27me3). Exposure times are given below each blot. Lanes R1, R2, and R3 represent biological replicates of *A. robustus*. +ve indicates positive control; left blot, rabbit anti-histone H3 antibody against *Candida glabarata*; middle blot, rabbit anti-H3K4me3 against *Candida glabarata*; right blot, rabbit anti-H3K27me3 against *Piromyces* sp. strain UH3-1. (B) Western blot images of *A. robustus* coculture with *F. succinogenes* strain UWB7 grown on Avicel with rabbit antibodies against histone 3 (H3), trimethylated lysine 4 on histone 3 (H3K4me3), and trimethylated lysine 27 on histone 3 (H3K27me3). Exposure times are given below each blot. Lanes R1, R2, and R3 represent biological replicates of *A. robustus*. +ve indicates positive control; left blot, rabbit anti-histone H3 antibody against *Candida glabarata*; middle blot, rabbit anti-H3K4me3 against *Candida glabarata*; right blot, rabbit anti-H3K27me3 against *Piromyces* sp. strain UH3-1. Replicates R2 and R3 are marked with red arrows for clarity in the middle blot. Download FIG S2, DOCX file, 0.7 MB. Copyright © 2021 Swift et al. 2021 Swift et al. https://creativecommons.org/licenses/by/4.0/ This content is distributed under the terms of the Creative Commons Attribution 4.0 International license.

To further understand the fungal regulatory response to cocultivation with *F. succinogenes* strain UWB7, we analyzed the eukaryotic orthologous groups (KOGs) (48) of the differentially regulated transcripts (Fig. S3). In the case of *A. robustus* cultured with *F. succinogenes* strain UWB7 on Avicel, the percentage of significantly downregulated fungal genes in coculture was greater than that upregulated for the majority of the KOG classes (absolute log_2_ fold change of ≥1, adjusted *P* value of <0.05). In other words, cocultivation repressed most fungal cellular and metabolic processes, with the exceptions of the KOG classes of posttranslational modifications and secondary metabolism. Table S4 lists the upregulated genes in coculture within the KOG class of secondary metabolism. In addition to the NRPS genes described above, 12 multidrug exporters belonging to the ABC superfamily were upregulated. The percentage of differentially regulated genes in all of the KOG classes was greater than 10%, with a median of 23% (Data Set S7 at the URL mentioned above). In contrast, cultivation on switchgrass resulted in far fewer differentially regulated genes in each KOG class between coculture and monoculture: *A. robustus* significantly regulated a median value of 1% of genes in each KOG class (Data Set S8 at the URL mentioned above), and *C. churrovis* regulated 5% (Data Set S9 at the URL mentioned above). These findings further support that the cultivation of *F. succinogenes* strain UWB7 with *A. robustus* on Avicel resulted in a stronger bacterial signal to the fungus. More broadly, it is clear that the choice of substrate, in addition to the specific organisms, has a profound impact on the gene regulation of microorganisms in cocultures.

10.1128/mBio.01442-21.5 TABLE S4 Upregulated genes in *A. robustus* (coculture with *Fibrobacter* UWB7 relative to *A. robustus* monoculture) assigned to the KOG class “Secondary metabolites biosynthesis, transport and catabolism.” All cultures were grown with Avicel as the substrate. Only genes with log_2_ fold change greater than one and adjusted *P* value less than 0.05 are shown. Genes on the same scaffold (boldface) are colocalized, immediate neighbors. The multidrug/pheromone exporter class is KOG0055, and the nonribosomal peptide synthetase KOG is KOG1178. Download Table S4, DOCX file, 0.01 MB. Copyright © 2021 Swift et al. 2021 Swift et al. https://creativecommons.org/licenses/by/4.0/ This content is distributed under the terms of the Creative Commons Attribution 4.0 International license.

10.1128/mBio.01442-21.8 FIG S3 Proportion of differentially expressed genes in each eukaryotic orthologous group (KOG) (48) class for anaerobic fungi in coculture with *Fibrobacter succinogenes* strain UWB7 relative to respective fungal monoculture. (A) *A. robustus* in coculture with *F. succinogenes* strain UWB7 on Avicel relative to *A. robustus* monoculture on Avicel (B) *A. robustus* in coculture with *F. succinogenes* strain UWB7 on switchgrass relative to *A. robustus* monoculture on switchgrass. (C) *C. churrovis* in coculture with *F. succinogenes* strain UWB7 on switchgrass relative to *C. churrovis* monoculture on switchgrass. KOG classes are organized into three plots for each comparison: cellular processes and signaling (top), information storage and processing (middle), and metabolism (bottom). Bar labels signify the percentage of genes upregulated (white) or downregulated (gray) in coculture. All carbohydrate active enzymes (CAZymes) with catalytic domains were binned into the KOG class carbohydrate transport and metabolism, and all CAZymes without a catalytic domain were excluded from this analysis. Download FIG S3, DOCX file, 0.2 MB. Copyright © 2021 Swift et al. 2021 Swift et al. https://creativecommons.org/licenses/by/4.0/ This content is distributed under the terms of the Creative Commons Attribution 4.0 International license.

### Rumen bacteria upregulate genes encoding components of drug efflux pumps when anaerobic fungi are present.

To further probe the relationship between *F. succinogenes* strain UWB7 and anaerobic gut fungi, we sequenced the corresponding prokaryotic mRNA in coculture and monoculture, using both eukaryotic and prokaryotic rRNA depletion (see Materials and Methods). When cocultivated with *C. churrovis* using switchgrass as the carbon source, *F. succinogenes* strain UWB7 upregulated 143 genes and downregulated 261 genes (4 and 8% of predicted genes in IMG/M [49]) (Data Set S10 at the URL mentioned above). Putative transporters comprised 12% of the upregulated genes. Table 2 summarizes upregulated genes encoding transporters (log_2_ fold change of ≥1.0, adjusted *P* value of <0.05). Notably, a predicted TolC family protein (locus tag Ga0136279_1901) was 2-fold upregulated in coculture relative to *F. succinogenes* strain UWB7 monoculture (adjusted *P* value of 1.5 × 10^−15^). TolC proteins are components of efflux pumps in Gram-negative bacteria, and these pumps can transport a wide array of molecules, including antibiotics (50). In addition, two genes encoding the adaptor subunits of RND efflux pumps (Ga0136279_1902 and Ga0136279_0657) were 2- and 3-fold upregulated in coculture (adjusted *P* value of ≤10^−6^), suggesting that they are part of a regulon. Upon inspection of the gene neighborhoods (Fig. S4), the TolC family protein encoded by Ga0136279_1901 and adapter subunit Ga0136279_1902 were neighboring genes. The coregulation of genes encoding components of multidrug efflux pumps has been previously reported. In *Enterobacteriaceae*, the genes encoding the multidrug efflux pump AcrAB-TolC (*acrA*, *acrB*, and *tolC*) form a regulon (51), although *tolC* is not colocalized with *acrA* and *acrB*. The 2-fold upregulated gene Ga0136279_2553, annotated as an ABC transporter substrate binding protein, also bordered a gene encoding a TolC family protein (Ga0136279_2554), although the gene encoding the putative TolC protein was not differentially regulated in coculture. For effective efflux directly into the external environment, both the outer membrane channel, such as TolC, and the periplasmic adaptor are necessary (52). Therefore, it is significant that both the TolC and adaptor protein homologs are upregulated when *F. succinogenes* strain UWB7 is cocultivated with *C. churrovis*.

**TABLE 2:** *F. succinogenes* UWB7 genes encoding putative transporters that were upregulated in coculture with *C. churrovis* relative to *Fibrobacter* monoculture^*b*^

Locus tag	Log_2_ fold change	Product name
Ga0136279_2636	2.9	ABC transporter ATP-binding protein
Ga0136279_2635	2.2	Putative ABC transport system permease protein
Ga0136279_2405	2.2	Type II and III secretion system protein
Ga0136279_1390	1.8	Outer membrane protein beta-barrel domain-containing protein
Ga0136279_1256	1.7	Urea ABC transporter substrate-binding protein
Ga0136279_0657	1.6	Multispecies efflux RND transporter periplasmic adaptor subunit
Ga0136279_2085	1.6	Zinc ABC transporter substrate-binding protein
Ga0136279_1465	1.5	Multispecies ammonium transporter
Ga0136279_2620	1.5	Transporter
Ga0136279_2553	1.4	Multispecies ABC transporter substrate-binding protein
Ga0136279_2080	1.4	Iron complex outer membrane recepter protein^*a*^
Ga0136279_1904	1.3	General secretion pathway protein E
Ga0136279_1405	1.2	TonB family C-terminal domain-containing protein^*a*^
Ga0136279_1902	1.1	Multispecies efflux RND transporter periplasmic adaptor subunit
Ga0136279_0818	1.0	TRAP transporter large permease subunit
Ga0136279_1391	1.0	Calcium/sodium antiporter
Ga0136279_1901	1.0	Multispecies TolC family protein

a Locus tags Ga0136279_2080 and Ga0136279_1405 are not transporters but are part of the TonB receptor complex involved in iron transport (83–85).

b Adjusted *P* value of less than 0.05. Product names were taken from the protein details for RefSeq NZ_FRCO00000000.1 or the gene product name in IMG/M (49).

10.1128/mBio.01442-21.9 FIG S4 Gene neighborhoods visualized using IMG/M (49) for Ga0136279_1902 (efflux RNA transporter periplasmic adapter subunit, log_2_ fold change of 1.1 for coculture versus monoculture) and Ga0136279_2080 (ABC transporter substrate binding protein, log_2_ fold change of 1.4 for coculture versus monoculture). Download FIG S4, DOCX file, 0.2 MB. Copyright © 2021 Swift et al. 2021 Swift et al. https://creativecommons.org/licenses/by/4.0/ This content is distributed under the terms of the Creative Commons Attribution 4.0 International license.

Besides drug efflux pumps, *F. succinogenes* strain UWB7 also upregulated at least 2-fold six genes encoding chaperones (Data Set S10 at the URL mentioned above), supporting the induction of a bacterial stress response by cocultivation with anaerobic fungi. Notably, *F. succinogenes* strain UWB7 also upregulated 32-fold a putative HicB antitoxin (Ga0136279_0693), which could be part of a toxin-antitoxin system (53). In addition, *F. succinogenes* strain UWB7 2-fold upregulated a gene encoding a putative abortive phage resistance protein (Ga0136279_0760), typically part of an RNA toxin-antitoxin system (54).

### Coculture of anaerobic fungi with bacteria points to the secretion of unique metabolites.

**FIG 4:**
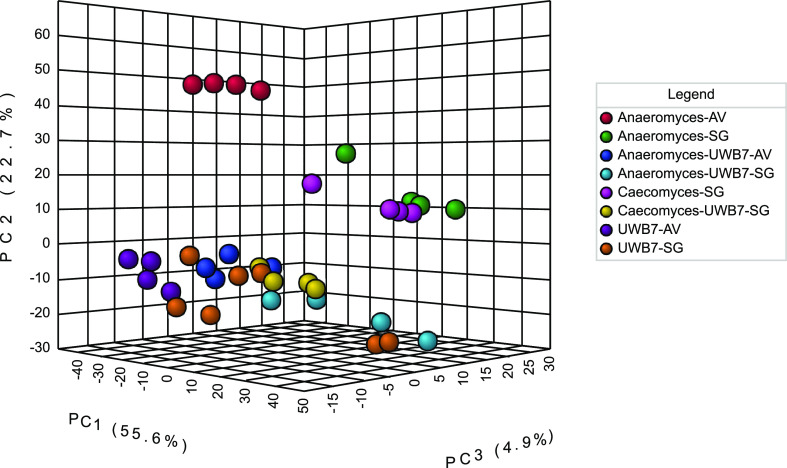
Metabolic profile of *A. robustus* cocultured with *F. succinogenes* strain UWB7 on Avicel is distinct from the respective fungal and bacterial monocultures. Three-dimensional principal-component analysis (PCA) scores plot of the untargeted nonpolar metabolomics data for cocultures and monocultures of *A. robustus*, *C. churrovis*, and *F. succinogenes* strain UWB7. AV, Avicel; SG, switchgrass. Plots were rendered by MetaboAnalyst (82).

To further test the hypothesis that cocultivation of anaerobic gut fungi with *F. succinogenes* strain UWB7 triggers the production of fungal defense compounds, we performed untargeted nonpolar metabolomics analysis on fungal-bacterial cocultures and the respective fungal or bacterial monocultures. We constructed a principal-component analysis (PCA) plot using MetaboAnalyst (55) (Fig. 4 and Fig. S5). In the three-dimensional scores plot (Fig. 4), bacterial monocultures and fungal-bacterial cocultures grown on switchgrass show a high degree of overlap. However, the cultures grown on Avicel (*A. robustus* monoculture, *F. succinogenes* strain UWB7 monoculture, and coculture) were distinct from each other. This separation is apparent in a three-dimensional PCA scores plot (Fig. 4) but not in the two-dimensional scores plot (Fig. S5). Taken together, these data suggest that, in contrast to cultures grown on switchgrass, the metabolic profiles observed in the cocultures of *A. robustus* with *F. succinogenes* strain UWB7 on Avicel are distinct from those observed in the respective monocultures.

10.1128/mBio.01442-21.10 FIG S5 Two-dimensional principal component analysis (PCA) plots of the untargeted nonpolar metabolomics data obtained for cocultures and monocultures of *A. robustus*, *C. churrovis*, and *F. succinogenes* strain UWB7. Empty symbols indicate monocultures, whereas symbols with interior crosses indicate cocultures. Ellipses represent 95% confidence regions. Plots were rendered by MetaboAnalyst (82). Download FIG S5, DOCX file, 0.2 MB. Copyright © 2021 Swift et al. 2021 Swift et al. https://creativecommons.org/licenses/by/4.0/ This content is distributed under the terms of the Creative Commons Attribution 4.0 International license.

**FIG 5:**
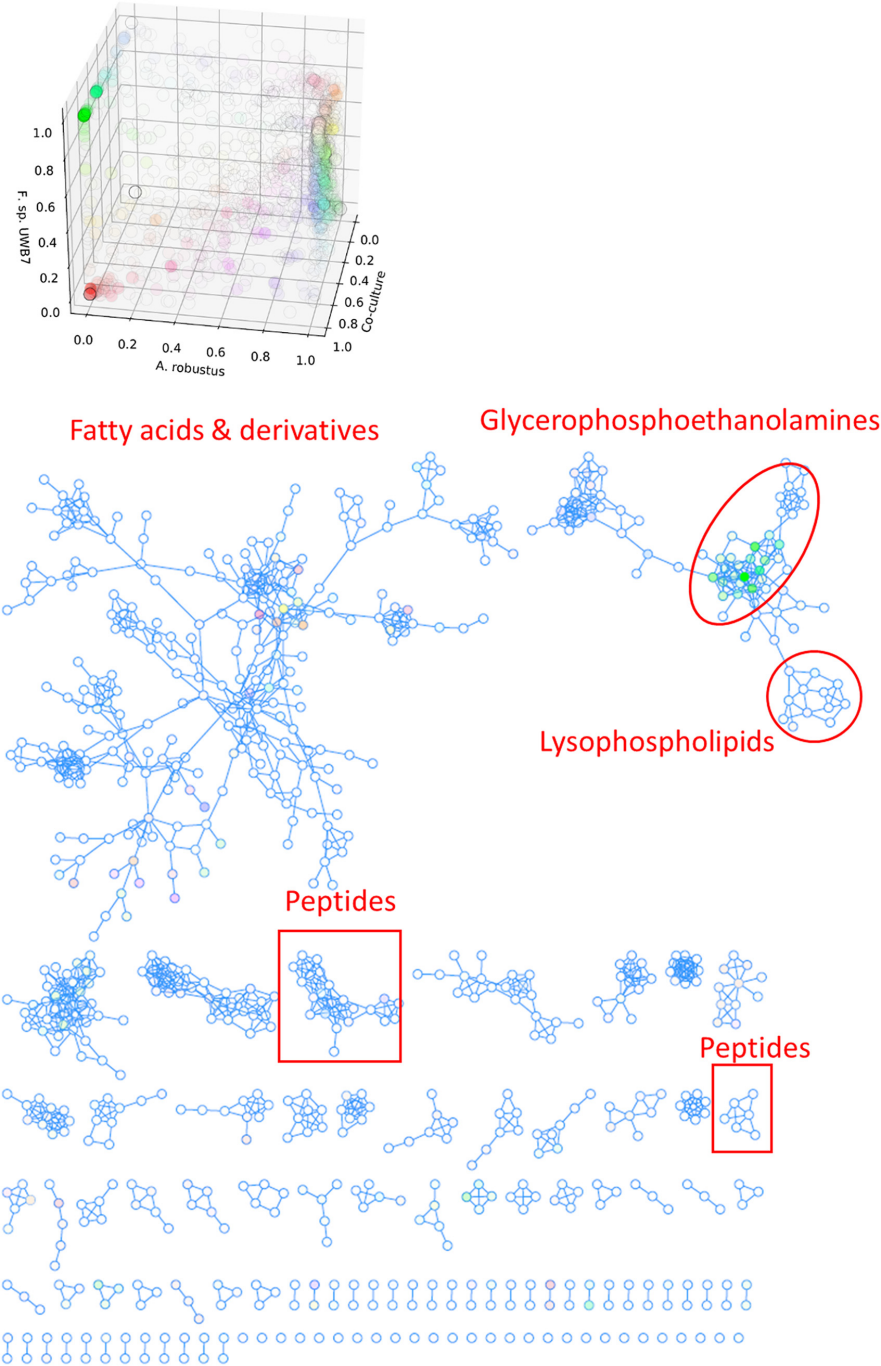
Cultivation of anaerobic fungi with *Fibrobacter succinogenes* strain UWB7 reveals diverse shared metabolites as well as a group of bacterial metabolites. Combined feature-based molecular network was created by GNPS (56) from positive- and negative-ion mode LC-MS/MS data from the nonpolar metabolites of *A. robustus*, *C. churrovis*, and *F. succinogenes* strain UWB7 cocultures and monocultures grown on Avicel or switchgrass substrates. Self-looping nodes were truncated. Three-way coloring (58) was used to visualize features in the *A. robustus* monocultures, *A. robustus*-*F. succinogenes* strain UWB7 cocultures, and *F. succinogenes* strain UWB7 monocultures (all grown on Avicel). Transparency of the nodes was set to emphasize nodes with high intensity in the coculture of *A. robustus*-*F. succinogenes* strain UWB7.

We further investigated the distribution of nonpolar metabolites between monocultures and cocultures by constructing molecular networks using Global Natural Products Social Molecular Networking (GNPS) (56) and visualizing the networks in Cytoscape (57) with three-way coloring (58) (Fig. 5). This approach highlighted a group of unknown metabolites unique to *F. succinogenes* strain UWB7 that were not observed in fungal monocultures. These metabolites were not enriched by cocultivation with anaerobic fungi and therefore likely represent constitutively produced bacterial metabolites. The closest known node in the cluster matched 1-palmitoyl-*sn*-glycero-3-phosphoethanolamine (*m/z* 454.2791), a lysophospholipid (LPL), which comprises a low concentration of Gram-negative bacterial cell membranes (59). Structure and molecular class predictions from SIRIUS (60) and CANOPUS (61) suggested that the unknown nodes represent glycerophosphoethanolamines. Overall this cluster of nodes likely represents components of the bacterial membrane that are released into the supernatant after cell death and lysis.

By comparing the peak height ratios of features between culture conditions, we searched for metabolites that were enriched by cocultivation with *F. succinogenes* strain UWB7. We screened these metabolites for those that were consistently observed across all four biological coculture replicates. We applied an enrichment threshold of 4-fold, reasoning that a 4-fold enhancement would most likely not be caused by the simple addition of bacterial and fungal biomass in coculture but rather increased production by one or both organisms. Four features matched these stringent criteria (Table 3). All four features are single nodes truncated from the molecular network depicted in Fig. 5 and, thus, are not likely to be part of a family of structurally related compounds. Notably, the feature *m/z* 244.227 was not observed in *F. succinogenes* strain UWB7 monoculture but was 12-fold enriched in coculture relative to *A. robustus* monoculture on Avicel, which suggests that this is a unique fungal metabolite with enhanced production in response to the presence of *F. succinogenes* strain UWB7.

**TABLE 3:** Metabolites enriched in fungal-bacterial cocultures relative to monocultures^*a*^

m/z	RT	Peak height fold change for:					
		Avicel (*A. robustus*)		Switchgrass			
				*A. robustus*		*C. churrovis*	
		Coculture/UWB7 monoculture	Coculture/fungal monoculture	Coculture/UWB7 monoculture	Coculture/fungal monoculture	Coculture/UWB7 monoculture	Coculture/fungal monoculture
196.040	2.39	12.89	19.97				
244.227	5.80	16.88	10.51	12.35	8.95		
380.277	4.98	—^*b*^	12.13				
408.308	7.43			4.52	13.20	4.98	4.96

a Untargeted nonpolar metabolomics features enriched at least 4-fold in coculture of *A. robustus* with *Fibrobacter succinogenes* strain UWB7 on Avicel compared to both bacterial and fungal monocultures (one-tailed Student’s *t* statistic, <0.05). All features were detected in positive-ion mode.

b —, feature was not detectable in UWB7 monoculture.

### Conclusions.

We have demonstrated using dual transcriptomics that, despite previous reports that Fibrobacter succinogenes had no interaction with rumen fungi (12, 14), as assessed by extent of biomass degradation in cocultures compared to monocultures, cocultivation of the close relative *F. succinogenes* strain UWB7 with *A. robustus* or *C. churrovis* resulted in drastic changes to both bacterial and fungal transcriptomes, including upregulation of bacterial drug efflux pumps and fungal chaperones, polyketide O-methyltransferases, PKSs, and NRPSs. Furthermore, fungal genes encoding putative histone-modifying enzymes were upregulated in coculture. Histone 3-lysine 27 trimethylation marks increased and heterochromatin-associated protein-1 was downregulated in coculture. Together, these results suggest that, similar to higher-order fungi, anaerobic fungi regulate their secondary metabolism via chromatin remodeling. These data support that anaerobic gut fungi activate their secondary metabolism via epigenetic and transcriptional regulation when challenged by rumen bacteria. The metabolic outcome of these transcriptional changes may be the production of a fungal defense compound produced by a PKS or NRPS. Consistent with this hypothesis, untargeted nonpolar metabolomics supports that at least one unique fungal metabolite is enriched by cocultivation with *F. succinogenes* strain UWB7. As a consequence, anaerobic fungi and the antagonistic relationships of the rumen microbiome may prove to be a valuable source of antibiotics.

## MATERIALS AND METHODS

### Isolation and cultivation of anaerobic gut fungi.

*Anaeromyces robustus* was isolated via reed canary grass enrichment from the fecal pellet of a Churro sheep at the Santa Barbara Zoo, as described previously (62, 63). *Caecomyces churrovis* was isolated similarly (64). Both fungi were cultivated anaerobically in Hungate tubes at 39°C with reed canary grass as the carbon source in a modified formulation (MC-) of complex medium C (65), containing 0.25 g/liter yeast extract (before boiling), 0.5 g/liter Bacto Casitone (before boiling), and 7.5 vol% clarified rumen fluid. The medium was supplemented with vitamins after autoclaving as described by Teunissen and colleagues (66). Cultures were passaged every 3 to 4 days into fresh media via a 1.0-ml sterile syringe.

### Cultivation of *F. succinogenes* strain UWB7.

The strain *F. succinogenes* strain UWB7 was a generous gift from Garret Suen at the University of Wisconsin-Madison. Details of the isolation of this strain are described in Neumann and Suen (28). *F. succinogenes* strain UWB7 was cultivated at 39°C anaerobically in Hungate tubes containing 9.0 ml of MC- medium supplemented with vitamin solution, prepared as described above, and 0.1 g of Avicel PH-101 (Sigma-Aldrich, St. Louis, MO). Each Hungate tube was inoculated with 1.0 ml of cryostock or live *F. succinogenes* strain UWB7 culture.

### Overview of the cocultivation conditions of anaerobic gut fungi with *Fibrobacter succinogenes* strain UWB7.

An overview of the cocultivation pairings, carbon substrates, and cocultivation incubation times is depicted in Fig. S1 in the supplemental material. Briefly, *A. robustus* was cocultivated with *Fibrobacter succinogenes* strain UWB7 on both Avicel PH-101 (Sigma-Aldrich, St. Louis, MO) and milled switchgrass (gift from U.S. Department of Agriculture), whereas *C. churrovis* was cocultivated with *F. succinogenes* strain UWB7 on switchgrass only due to the slow growth of *C. churrovis* on Avicel (64). Since *A. robustus* and *C. churrovis* are expected to grow more slowly than *F. succinogenes* strain UWB7, as evidenced by the order-of-magnitude larger specific growth rate of *F. succinogenes* compared to *C. churrovis* on soluble sugars (64, 67), both strains of anaerobic gut fungi were allowed to grow for 24 h prior to inoculation with *F. succinogenes* strain UWB7. Cocultures were subsequently allowed to grow for an additional 24 to 72 h prior to harvesting for RNA extraction. The length of incubation for the cocultivation pairings was set by time necessary for the fungus to reach mid-log growth phase, as assessed by cumulative pressure (68) of fungal monocultures as well as visual assessment.

### Cocultivation of anaerobic fungi with *Fibrobacter succinogenes* strain UWB7.

Anaerobic liquid growth medium MC- was prepared by following the recipe for complex medium C (65), with yeast extract, Bacto Casitone, and clarified rumen fluid reduced to 0.25 g/liter (concentration before boiling), 0.5 g/liter (before boiling), and 7.5 vol%, respectively. A 100-ml capacity serum bottle was filled with 80 ml of MC- liquid medium and 0.8 g of switchgrass or Avicel PH-101 (Sigma-Aldrich, St. Louis, MO). The serum bottle and its contents were flushed with CO_2_, autoclaved, and supplemented with 0.8 ml of 100× vitamin solution (66). The serum bottle was preheated to 39°C, and then a seed culture of *A. robustus* was started by inoculating 1.0 ml of the routinely passaged *A. robustus* described above into the liquid medium using a 1-ml sterile syringe. The seed culture was immediately vented following inoculation and then incubated at 39°C for 4 days. This seed culture was used to inoculate cultures to be harvested for subsequent RNA-seq. Cultures were prepared in replicates of four by inoculating 1.0 ml of *A. robustus* into 8.0 ml (coculture) or 9.0 ml (monoculture) of MC- containing 0.1 g switchgrass or Avicel PH-101 (Sigma-Aldrich, St. Louis, MO). Prior to inoculation, the medium and substrate were autoclaved, supplemented with 0.1 ml of 100× vitamin solution (66) postautoclaving, and preheated to 39°C. The fungal culture was grown at 39°C for 24 h, and then cocultures were started by inoculating 1.0 ml of *F. succinogenes* strain UWB7 into each of four replicates. The seed culture of *F. succinogenes* strain UWB7 was grown for 24 h at 39°C from 1.0 ml of inoculum in a serum bottle containing 80 ml of MC-, 0.8 g of Avicel PH-101 (Sigma-Aldrich, St. Louis, MO), 0.8 ml of 100× vitamin solution (66), and a CO_2_ headspace. *A. robustus* monocultures and *A. robustus*-*F. succinogenes* strain UWB7 cocultures were incubated for an additional 24 h (switchgrass substrate) or 48 h (Avicel). The contents of each Hungate tube were transferred to 15-ml Falcon tubes (Fisher Scientific, Waltham, MA) and centrifuged at 3,200 × *g* and 4°C using a swinging-bucket rotor (Eppendorf A-4-81) for 10 min. The supernatant was saved at −80°C for subsequent liquid chromatography-tandem mass spectrometry (LC-MS/MS), and 1.0 ml of RNAlater (Sigma-Aldrich, St. Louis, MO) was added to the pellet to preserve the RNA. The pellet from each culture was frozen at −80°C until lysis.

The cocultivation of *C. churrovis* with *F. succinogenes* strain UWB7 was performed identically to the cocultivation of *A. robustus* with *F. succinogenes* strain UWB7, except that the length of the cocultivation incubation was 40 h.

### RNA extraction and QC.

Samples were thawed on ice and then centrifuged at 3,220 × *g* at 4°C using a swinging-bucket rotor (Eppendorf A-4-81) for 10 min. RNAlater was decanted. The pellets were transferred into 2-ml screw-cap tubes containing 1.0 ml of autoclaved 0.5-mm zirconia/silica beads (Biospec) and 600 μl buffer RLT (Qiagen, Hilden, Germany) with 1 vol% 2-mercaptoethanol (Sigma-Aldrich, St. Louis, MO). The tube was briefly vortexed, and then the cells were lysed using the Biospec Mini-Beadbeater-16 for 45 s. The tubes were placed on ice for 30 s. Following lysis, the tubes were centrifuged for 3 min at 13,000 × *g* and 20°C using a microcentrifuge (Eppendorf 5424). The supernatant was removed using gel-loading tips (Fisher Scientific, Waltham, MA) to maximize yields and centrifuged again to remove residual cell debris for 2 min at 20,000 × *g*. The supernatant from each tube was then transferred into 2-ml round-bottom sample tubes (Qiagen catalog number 990381). RNA was extracted using a QIAcube by following the RNeasy Mini protocol for animal cells with QIAshredder homogenization and optional on-column DNase digest.

Quantity and quality of RNA was assessed by a QuBit fluorometer and TapeStation (Agilent), respectively. All RNA integrity number equivalents (RIN^e^) were above 6.0, assessed by either eukaryotic or prokaryotic ribosomal markers for cocultures.

### RNA library preparation and sequencing.

In fungal monocultures, fungal mRNA was selectively enriched by capturing polyadenylated RNA using poly-T beads. For bacterial monocultures, rRNA was depleted using the Illumina Ribo-Zero rRNA removal kit (Yeast) spiked into the Illumina Ribo-Zero gold rRNA removal kit (Epidemiology). To obtain both bacterial and fungal libraries from the cocultures, each sample was divided, and 200 ng was used as the input into each alternative pipeline: (i) poly(A) selection for the fungal library or (ii) ribosomal depletion by an Illumina Ribo-Zero rRNA removal kit (yeast) spiked into the Illumina Ribo-Zero gold rRNA removal kit (Epidemiology) for the library enriched in bacterial mRNA. Stranded RNA-seq libraries were created by the Joint Genome Institute and quantified by quantitative PCR (qPCR). Libraries were sequenced by paired-end 150-bp reads using a NovaSeq (Illumina, San Diego, CA).

### RNA-seq data analysis.

Raw reads were evaluated for artifact sequences using BBDuk (69). Detected artifacts identified using kmer matching (kmer = 25) were trimmed from the 3′ end of reads. Reads were further filtered by removing RNA spike-in reads, PhiX reads, and reads containing any N’s. Reads were trimmed for quality using the phred trimming method (set at Q6). Following trimming, short reads of less than 50 bases were removed. Filtered reads were aligned to the reference genome for the respective organism (fungal genomes available on the MycoCosm portal [30] and *F. succinogenes* strain UWB7 genome GenBank assembly accession no. GCA_900142945.1) using HISAT2 version 2.1.0 (70). Raw gene counts were generated by featureCounts (71) using the gene annotation files available in MycoCosm (30) for *A. robustus* or *C. churrovis* and IMG (49) for *Fibrobacter* sp. strain UWB7.

The effectiveness of poly(A) selection and ribosomal depletion methods was quantified using SortMeRNA (72) and is discussed in Text S1 in the supplemental material. Differential expression analysis was performed using DESeq2 (version 1.18.1) (73), with a minimum absolute log_2_ fold change of 1.0 and statistical significance threshold of adjusted *P* value of less than 0.05 (Benjamini and Hochberg method). Gene set enrichment analysis (27) was performed with regard to gene sets comprised of eukaryotic orthologous group (48) KOG0710, molecular chaperones from the small heat shock protein Hsp20/Hsp42 family. GSEAPre-ranked was used with the log_2_ fold change of cocultures to monocultures (output from DESeq2) as the input in RNK format. The following parameters were used: the number of permutations was set to 1,000, and maximum and minimum sizes of gene sets to exclude were set to 500 and 5, respectively.

10.1128/mBio.01442-21.1 TEXT S1 Evaluation of poly(A) selection and ribosomal depletion effectiveness in cocultures and transcriptomic analysis of fungal and bacterial carbohydrate active enzymes in coculture compared to monoculture. Download Text S1, DOCX file, 0.02 MB. Copyright © 2021 Swift et al. 2021 Swift et al. https://creativecommons.org/licenses/by/4.0/ This content is distributed under the terms of the Creative Commons Attribution 4.0 International license.

### Extraction and LC-MS/MS.

Two milliliters of ethyl acetate was added to 1.5 ml of fungal supernatant, vortexed, sonicated for 10 min in a water bath (room temperature), and centrifuged (5 min at 5,000 rpm), and then the top ethyl acetate layer was removed to another tube. To serve as extraction controls, tubes without sample were extracted by following the same procedure. Extracts were dried in a SpeedVac (SPD111V; Thermo Scientific) and stored at −80°C.

In preparation for LC-MS analysis, 150 μl LC-MS-grade methanol containing 1 μg/ml internal standard (2-amino-3-bromo-5-methylbenzoic acid; Sigma) was added to dried extracts, followed by a brief vortex and sonication in a water bath for 10 min; 150 μl of resuspended extract was then centrifuge filtered (2.5 min at 2,500 rpm) using a 0.22-μm filter (UFC40GV0S; Millipore) and transferred to a glass autosampler vial. Reverse-phase chromatography was performed by injecting 2 μl of sample into a C_18_ chromatography column (2.1 by 50 mm, 1.8 μm; Agilent ZORBAX Eclipse Plus C_18_) warmed to 60°C with a flow rate of 0.4 ml/min equilibrated with 100% buffer A (100% LC-MS water with 0.1% formic acid) for 1 min, followed by a linear gradient to 100% buffer B (100% acetonitrile with 0.1% formic acid) for 7 min and then held at 100% B for 1.5 min. MS and MS/MS data were collected in both positive- and negative-ion mode using a Thermo Q Exactive HF mass spectrometer (ThermoFisher Scientific, San Jose, CA), with full MS spectra acquired ranging from 80 to 1,200 *m/z* at 60,000 resolution, and fragmentation data were acquired using an average of stepped collision energies of 10, 20, and 40 eV at 17,500 resolution. Orbitrap instrument parameters included a sheath gas flow rate of 50 (au, arbitrary units), auxiliary gas flow rate of 20 (au), sweep gas flow rate of 2 (au), 3-kV spray voltage, and 400°C capillary temperature. Sample injection order was randomized and an injection blank of methanol only run between each sample. Raw data are available for download at https://genome.jgi.doe.gov/portal/ under the JGI Project ID 1294405.

### Metabolomics data analysis: creation of molecular network via Global Natural Products Social Molecular Networking.

The Feature-Based Molecular Networking (FBMN) workflow (74) on GNPS (56) (https://gnps.ucsd.edu) was used to construct a molecular network. First, peak finding was performed with MZmine (version 2.39) (75). An MZmine workflow was used to generate a list of features (*m/z*, residence time values obtained from extracted ion chromatograms containing chromatographic peaks within a narrow *m/z* range) and filtered to remove isotopes, adducts, and features without MS/MS. ADAP chromatogram builder and deconvolution modules were used (76). The exact parameters used are available in an XML document upon request from the corresponding author. This document describes the batch operations that can be read by MZMine directly. The molecular networking GNPS job can be publicly accessed at https://gnps.ucsd.edu/ProteoSAFe/status.jsp?task=aeeb3b1a8fac4b67b54b6f1171a3053f.

For each feature, the most intense fragmentation spectrum was uploaded to GNPS. All MS/MS fragments were removed within ±17 Da of the precursor *m/z*. Window filtering was achieved by selecting only the top 6 fragment ions in the ±50-Da window throughout the spectrum. Parameters were set as a precursor ion mass tolerance of 0.05 Da and MS/MS fragment ion tolerance of 0.05 Da. The edges of the molecular network were specified to have a cosine score greater than 0.70 and more than 6 matching peaks. The edges were further filtered such that an edge was permitted if and only if the joined nodes were present in the other respective node’s top 10 most similar nodes. Lowest-scoring edges were removed from molecular families such that no family contained more than 100 nodes. All spectra within the molecular network were queried against GNPS spectral libraries (56). Each library spectrum was filtered by following the same procedure as that applied to the input data. The minimum criteria for a match between a network spectrum and a library spectrum were that the score be greater than 0.7 and that at least 6 peaks match. MS/MS spectra were annotated by DEREPLICATOR (77).

It should be noted that a spectrum match to a database spectrum is not a definitive identification of the feature. It could be an isomer with a similar fragmentation, an ion with a close but not exact *m/z* but similar fragmentation pattern, or an in-source degradation product of another larger molecule (the degradation product may look similar to the database match).

GNPS positive- and negative-mode networks were merged using a custom Python script to group nodes having a retention time difference of less than 0.15 min and an *m/z* difference of less than 20 parts per million, assuming the negative mode species ionized as [M-proton]^−^ and the positive mode species ionized as [M+proton]^+^. The resulting network is available as a pdf (Data Set S11 at https://github.com/cswift3/anaerobic_fungi_Fibrobacter_co-culture) or in GRAPHML format (for direct visualization in Cytoscape [57]) upon request from the corresponding author.

Finally, the molecular network was visualized using Cytoscape (57) and three-way coloring. Given three numerical values to compare, the corresponding hue for each value can be calculated according to Baran and colleagues (58) using a custom Python script. The transparency of each node is determined by the value of each normalized to the minimum and maximum of the set of values. In this case, the three values to compare were the GNPS-normalized peak areas of each feature (averaged across four biological replicates) for three different treatments: (i) *A. robustus* monoculture (Avicel substrate), (ii) *F. succinogenes* strain UWB7 monoculture (Avicel substrate), and (iii) *A. robustus*-*F. succinogenes* strain UWB7 coculture (Avicel substrate). The method of per-sample normalization selected in the GNPS job was “row sum normalization (per file sum to 1,000,000),” and the mean was chosen as the aggregation method per group (treatment). For the molecular network depicted in Fig. 5, the transparency of each node was normalized with respect to the minimum and maximum GNPS-normalized peak areas of the coculture condition. For example, features that are many orders of magnitude more intense in one group than another will not be transparent (high alpha). A square root normalization was applied to the intensity difference to calculate transparency values that emphasize the most important features. In comparison, features that have approximately the same intensity in all treatment groups will have high transparency (low alpha).

Structure and class prediction of the unknown bacterial metabolites (glycophosphoethanolamines; Fig. 5) was performed by SIRIUS 4.0 (60) and CANOPUS (61) by the MS-GF+ (1.3.0) workflow on the ProteoSAFe web server from the Center for Computation Mass Spectrometry. The job may be viewed and cloned from https://proteomics2.ucsd.edu/ProteoSAFe/status.jsp?task=e49148e8624c4e4cb0c3fbe09918ab6c.

### PCA of metabolomics data using MetaboAnalyst 4.0.

MetaboAnalyst 4.0 (78) was used to construct principal-component analysis (PCA) plots from the peak heights feature table of all samples, generated using MZmine2 (75, 79). Sample normalization was set to “normalization by sum.” The “prcomp” function in R (80), which requires the package “chemometrics,” was used internally within MetaboAnalyst to perform the PCA.

### Growth of fungal cultures for Western blotting of fungal epigenetic modifications.

To prepare the *A. robustus* seed culture, 1.0 ml of *A. robustus* from routine cultivation was transferred into a 60-ml glass serum bottle preheated to 39°C containing 40 ml of MC- medium with 0.4 g of Avicel PH-101 (Sigma-Aldrich, St. Louis, MO) and supplemented with 0.4 ml of 100× vitamin solution (66) after autoclaving. The seed culture was grown for 4 days. From the seed culture, 1.0 ml was inoculated into each of six 80-ml cultures of MC- with 0.8 g Avicel PH-101 (Sigma-Aldrich, St. Louis, MO) supplemented with 0.8 ml of 100× vitamin solution (66). Three of these cultures were incubated at 39°C for 24 h, and then each bottle was inoculated with 1.0 ml of *F. succinogenes* strain UWB7 seed culture. The remaining three bottles were also incubated at 39°C, but they were not inoculated with *F. succinogenes* strain UWB7. The *F. succinogenes* strain UWB7 seed culture was prepared by inoculating 1.0 ml of active culture into a 60-ml serum bottle containing 40 ml of MC- supplemented with vitamin solution (66) and 0.4 g of Avicel PH-101 (Sigma-Aldrich, St. Louis, MO). The active culture was *F. succinogenes* strain UWB7 revived from cryostock 1 week prior and passaged one time. The cocultures and monocultures were grown for a total of 72 h following the fungal inoculation. The cultures were then transferred into 50-ml Falcon tubes and centrifuged using a fixed-angle rotor (Eppendorf F-34-6-38) at 4°C and 3,000 × *g* for 10 min. The cell pellets were stored at −80°C until lysis.

### Extraction of fungal cultures for Western blotting of fungal epigenetic modifications.

The frozen cell pellets prepared above were resuspended in 3 ml of 2 M NaOH (Fisher Scientific, Waltham, MA, USA) with 10% (vol/vol) beta-mercaptoethanol (Sigma-Aldrich, St. Louis, MO, USA). The solution was gently mixed and incubated on ice for 5 min to promote hydrolysis of the fungal cell wall. The solution was then centrifuged at 14,000 × *g* for 30 s at 4°C. The resulting pellet was resuspended in 3 ml of high-salt extraction buffer containing 40 mM HEPES, pH 7.5 (Fisher Scientific, Waltham, MA, USA), 350 mM NaCl (Fisher Scientific, Waltham, MA, USA), 0.1% (wt/vol) Tween 20 (Bio-Rad, Hercules, CA, USA), and 10% (vol/vol) glycerol (Fisher Scientific, Waltham, MA, USA). The solution was immediately centrifuged at 14,000 × *g* for 30 s at 4°C. The cell pellets were resuspended in 3 ml of 2× SDS sample buffer, 0.1 M Tris-HCl, pH 6.8 (Fisher Scientific, Waltham MA, USA), 4% (wt/vol) SDS (Fisher Scientific, Waltham, MA, USA), 0.2% (wt/vol) bromophenol blue (Sigma-Aldrich, St. Louis, MO, USA), 20% (vol/vol) glycerol (Fisher Scientific, Waltham, MA, USA), and 10% (vol/vol) beta-mercaptoethanol (Sigma-Aldrich, St. Louis, MO, USA). Cell pellets incubated at 100°C for 10 min, prior to being centrifuged at 14,000 × *g* for 30 s at 4°C. The supernatants were stored at −20°C until further use.

### Western blotting of fungal epigenetic modifications.

Thirty microliters of previously frozen cell lysates prepared in SDS sample buffer was gently mixed prior to loading on a 15% polyacrylamide gel. Candida glabrata whole-cell lysate, extracted as mentioned for the extraction of fungal cultures for Western blotting, was used as the histone H3 nuclear loading control as well as a positive control for H3K4me3 Western blots. *Piromyces* sp. strain UH3-1 whole-cell lysate was used as a loading control for H3K27me3 Western blots. Gel electrophoresis occurred for 65 min at 150 V under constant voltage at room temperature. The gel and Immobilon polyvinylidene difluoride (PVDF) membrane (Fisher Scientific, Waltham, MA, USA) were briefly washed with 100% methanol (Fisher Scientific, Waltham, MA, USA) prior to being washed with 1× Towbin buffer containing 25 mM Tris, pH 8.3 (Thomas Scientific, Swedesboro, NJ, USA), 192 mM glycine (Sigma-Aldrich, St. Louis, MO, USA), and 10% (vol/vol) methanol. The gel and membrane were overlaid on top of 9 pieces of 3MM chromatography paper (Fisher Scientific, Waltham, MA, USA) that were soaked in 1× Towbin buffer. After overlaying the gel and membrane on top of the 9 sheets of chromatography paper, an additional six sheets of chromatography paper already saturated with 1× Towbin buffer were overlaid on top of the gel. Both membrane and chromatography paper were previously cut to dimensions of 5.5 cm by 8.5 cm in order to match the dimensions of the resolving gel. After rolling out the transfer sandwich to remove air bubbles, the proteins were transferred under semidry conditions using a Hoefer Hsi Semi-phor TE70 semidry transfer unit (Holliston, MA, USA) for 90 min at 42 mA under constant amperage at room temperature. Membranes were blocked overnight with 3% (wt/vol) milk (Great Value, Bentonville, AR, USA) with 0.15% (wt/vol) sodium azide (Sigma-Aldrich, St. Louis, MO, USA) dissolved in 1× Tris-buffered saline (TBS), pH 7.5 (Fisher Scientific, Waltham, MA, USA), at 4°C. The following day, membranes were washed at room temperature for 30 min with 1× TBS buffer, pH 7.5, exchanging the buffer every 10 min. Primary antibodies were diluted in 10 ml of 1× TBS buffer and incubated with the membranes for approximately 3 h at room temperature on a rocker at slow speed. Rabbit anti-histone H3 antibody (ab1791; Abcam, Cambridge, MA, USA) diluted 1:10,000 in 1× TBS buffer was used as a nuclear loading control. Rabbit anti H3K4me3 antibody (39016; Activemotif, Carlsbad, CA, USA) was diluted 1:50,000 in 1× TBS buffer. Rabbit anti-H3K27me3 antibody (07-449; Upstate-Millipore, Lake Placid, NY, USA) was diluted 1:5,000 in 1× TBS, pH 7.5. After 3 h, the blots were washed with 1× TBS, pH 7.5, for 30 min, exchanging the buffer every 10 min. Horseradish peroxidase-conjugated goat anti-rabbit secondary antibody (111-035-003; Jackson ImmunoResearch, West Grove, PA, USA) was diluted 1:10,000 in 1× TBS, pH 7.5, and 10 ml of this solution was added to each blot, which was incubated on a rocker for 3 h at room temperature. The blots were washed with 1× TBST buffer (10 mM Tris, pH 7.5, 100 mM NaCl, 0.1% [wt/vol] Tween 20) for 30 min, exchanging the buffer every 10 min. A volume of 300 μl of Crescendo horseradish peroxidase reagent (Fisher Scientific, Waltham, MA, USA) was added to each blot, and the blots were imaged in a Bio-Rad Chemidoc imager under default chemiluminescence settings and autoadjusted exposure time. For the H3 blots, the positive loading control was masked during autoadjusted exposure to avoid overwhelming the sample signals.

### Helium ion microscopy.

The *A. robustus*-*F. succinogenes* strain UWB7 coculture was prepared as described above. The cell pellet, including the Avicel growth substrate, was harvested and suspended in phosphate-buffered saline (PBS, pH 7.5) in a 15-ml Falcon tube, to which glutaraldehyde (Sigma-Aldrich, St. Louis, MO) was added to a final concentration of 2 vol%. The tubes were incubated at room temperature for 1 h on a rotator. The tubes were then centrifuged at 700 × *g* for 10 min at 4°C, and the buffer was removed. The pellet was resuspended in 10 ml of 25 vol% ethanol and incubated for another hour. This process of suspension, incubation, and centrifugation was repeated for a stepwise ethanol dehydration series with 30%, 50%, and 70% ethanol steps. Twice more the pellet was washed with 10 ml of 100% ethanol, incubated for 15 min, and finally resuspended in 5 ml of 100% ethanol. The cells were then dried via critical point drying with an Autosamdri-815 (Tousimis, Rockville, MD) and carbon dioxide as a transitional fluid, sputter-coated with conductive carbon, and imaged using an Orion helium ion microscope (Carl Zeiss Microscopy, Peabody, MA).

### Data availability.

Supplementary data sets are available at the following Github repository: https://github.com/cswift3/anaerobic_fungi_Fibrobacter_co-culture. All sequencing reads have been deposited in the Sequencing Read Archive (SRA) and are associated with NCBI BioProject PRJNA666900. The raw mass spectrometry data were deposited on the MassIVE public repository (MSV000086033). Raw data are also available for download at https://genome.jgi.doe.gov/portal/ under the JGI Project ID 1294405. The molecular networking GNPS job can be publicly accessed at https://gnps.ucsd.edu/ProteoSAFe/status.jsp?task=aeeb3b1a8fac4b67b54b6f1171a3053f. The ProteoSAFE job may be viewed and cloned from https://proteomics2.ucsd.edu/ProteoSAFe/status.jsp?task=e49148e8624c4e4cb0c3fbe09918ab6c.
