# Supplementary material for: Cocultivation of Anaerobic Fungi with Rumen Bacteria Establishes an Antagonistic Relationship
Source: mBio. 2021 Aug 17;12(4):e01442-21. doi: 10.1128/mBio.01442-21 (PMC8406330; doi:10.1128/mBio.01442-21)
Supplement: FIG S1 [file mbio.01442-21-sf001.docx]

**(A)**


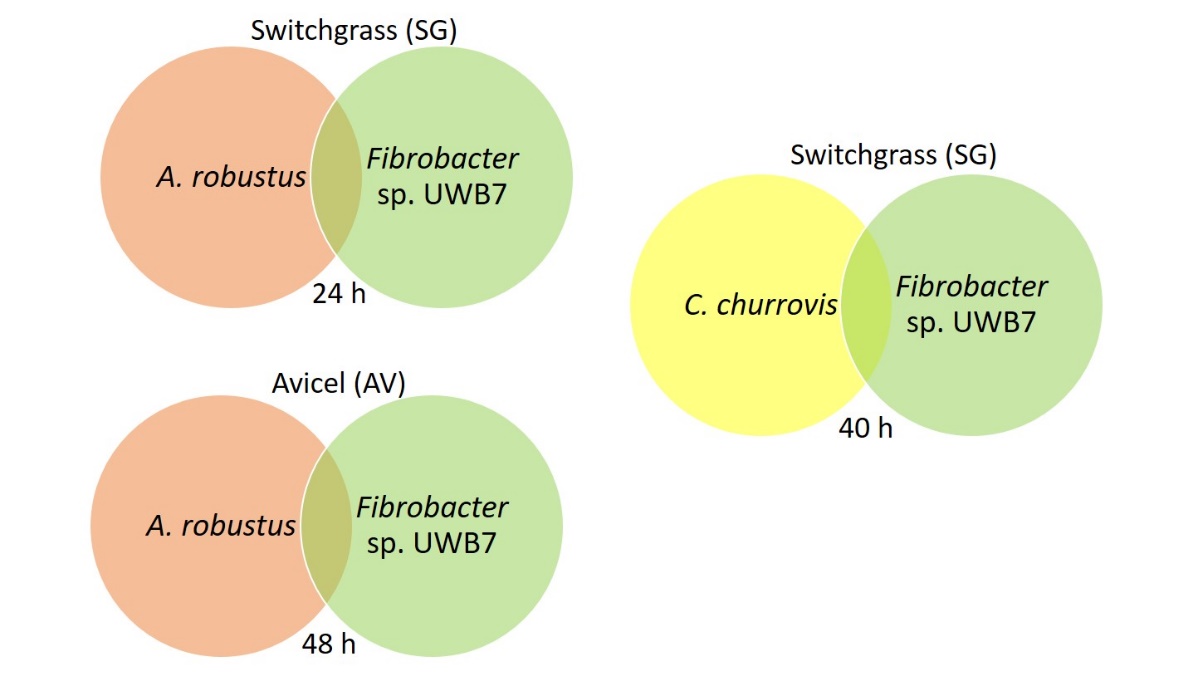


**(B)**

**
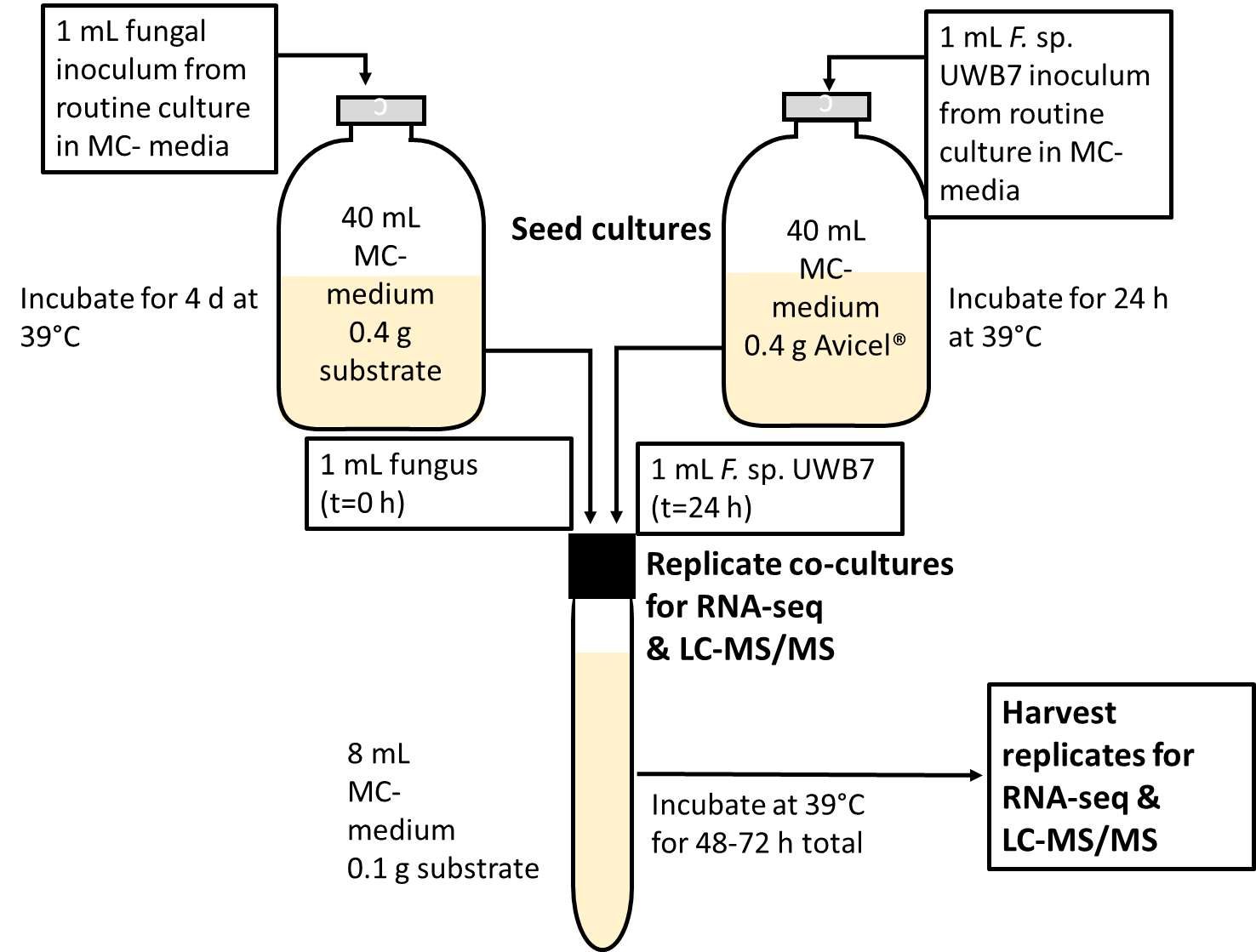
**

**Supplementary Figure S1.** Experimental design of fungal-bacterial co-cultures. (A) Schematic of the co-cultivation pairings of anaerobic fungi with the rumen bacteria *F*. sp. UWB7 on two different carbon substrates. Fungal cultures were grown for 24 h prior to the introduction of *F*. sp. UWB7 and subsequently co-cultured for the duration listed below each Venn diagram. (B) Detailed experimental schematic of the preparation of cultures for transcriptomics and metabolomics. Seed cultures of fungus and *F.* sp. UWB7 were inoculated into serum bottles and grown for 4 days (fungus) or 24 hours (bacteria). These cultures were used as the inoculum for Hungate tubes (4 biological replicates per condition), which were subsequently harvested after at least 48 hours of growth post inoculation. For co-cultures, the fungus was grown in isolation for 24 hours prior to the introduction of bacteria.
