# Supplementary material for: Cocultivation of Anaerobic Fungi with Rumen Bacteria Establishes an Antagonistic Relationship
Source: mBio. 2021 Aug 17;12(4):e01442-21. doi: 10.1128/mBio.01442-21 (PMC8406330; doi:10.1128/mBio.01442-21)
Supplement: TABLE S3 [file mbio.01442-21-st003.docx]

**Supplementary Table S3.** Anaerobic gut fungi *A. robustus* and *C. churrovis* upregulate genes with potential functions in secondary metabolism or chromatin remodeling when co-cultures with *F.* sp. UWB7. Genes with putative functions in secondary metabolism include those involved in polyketide and non-ribosomal peptide synthesis as well as SAM-dependent methyltransferases that are potentially involved in the regulation of secondary metabolism. Adjusted *p*-value less than 0.05 for all comparisons.

| MycoCosm protein Id | InterPro Id | Log2fold change | | Product name or InterPro annotation | |  |
| --- | --- | --- | --- | --- | --- | --- |
| ***A. robustus* (Avicel^®^)** | | | | | |  |
| **Genes with potential functions in secondary metabolism** | | | | |  |  |
| 298422 | IPR016874 | 5.1 | Polyketide synthesis O-methyltransferase | |  |  |
| 325735 | IPR003455, IPR016874 | 4.1 | Polyketide synthesis O-methyltransferase | |  |  |
| 325736 | IPR003455 | 3.2 | O-methyltransferase domain protein | |  |  |
| 325734 | IPR003455, IPR16874 | 3.0 | Polyketide synthesis O-methyltransferase | |  |  |
| 266064 | IPR003455, IPR16874 | 2.7 | Polyketide synthesis O-methyltransferase | |  |  |
| 297314 | IPR013217 | 1.4 | S-adenosyl-L-methionine-dependent methyltransferase | |  |  |
| 271870 | IPR001242 | 1.2 | Condensation domain | |  |  |
| **Genes with potential functions in chromatin remodeling** | | | | |  |  |
| 328372 | IPR013216 | 5.4 | Methyltransferase type 11 | |  |  |
| 325551 | IPR013216 | 2.0 | Methyltransferase type 11 | |  |  |
| 266199 | IPR000182, IPR016181 | 2.0 | Acyl-CoA N-acyltransferase | |  |  |
| 47572 | IPR013216 | 1.9 | Methyltransferase type 11 | |  |  |
| ***C. churrovis* (switchgrass)** | | | | | |  |
| **Genes with potential functions in secondary metabolism** | | | | | | |
| 525731 | IPR007213, IPR016874, IPR029063 | 4.1 | S-adenosyl-L-methionine-dependent methyltransferase | | | |
| 619287 | IPR007213, IPR029063 | 3.8 | Leucine carboxyl methyltransferase-domain containing protein | | | |
| 581095 | IPR007213, IPR029063 | 3.5 | S-adenosyl-L-methionine-dependent methyltransferase | | | |
| 270385 | IPR007213, IPR016874, IPR029063 | 3.4 | S-adenosyl-L-methionine-dependent methyltransferase | | | |
| 175508 | IPR007213, IPR016874, IPR029063 | 3.0 | Putative tetracenomycin polyketide synthesis O-methyltransferase | | | |
| 462789 | IPR007213, IPR016874, IPR029063 | 2.5 | S-adenosyl-L-methionine-dependent methyltransferase | | | |
| **Genes with potential functions in chromatin remodeling** | | | | | | |
| 619316 | IPR013216, IPR025714, IPR029063 | 3.7 | Methyltransferase type 11 | | | |
| 451944 | IPR013216, IPR029063 | 2,3 | Methyltransferase type 11 | | | |
| 627120 | IPR000181, IPR000182 | 2.2 | Acyl-CoA N-acyltransferase | | | |
| 479563 | IPR013216, IPR029063 | 2.0 | Methyltransferase type 11 | | | |
| 522030 | IPR000182 | 1.4 | Acyl-CoA N-acyltransferase | | | |
| 78835 | IPR00181, IPR000182 | 1.1 | Acyl-CoA N-acyltransferase | | | |
