# Supplementary material for: Cocultivation of Anaerobic Fungi with Rumen Bacteria Establishes an Antagonistic Relationship
Source: mBio. 2021 Aug 17;12(4):e01442-21. doi: 10.1128/mBio.01442-21 (PMC8406330; doi:10.1128/mBio.01442-21)
Supplement: TABLE S1 [file mbio.01442-21-st001.docx]

**Supplementary Table S1**. Assessment of the effectiveness of ribosomal depletion in sequenced libraries of co-cultures of *C. churrovis* and *F.* sp. UWB7 and monocultures of *F.* sp. UWB7 grown on switchgrass. Reads were categorized by SortMeRNA(2) with reference to the SILVA (3) and Rfam (4) databases. Read percentages are based on quality-filtered reads as described in the methods.
