# Supplementary material for: Cocultivation of Anaerobic Fungi with Rumen Bacteria Establishes an Antagonistic Relationship
Source: mBio. 2021 Aug 17;12(4):e01442-21. doi: 10.1128/mBio.01442-21 (PMC8406330; doi:10.1128/mBio.01442-21)
Supplement: TABLE S2 [file mbio.01442-21-st002.docx]

**Supplementary Table S2.** Number of differentially regulated fungal genes in co-culture with *F.* sp. UWB7 compared to fungal monoculture (absolute log_2_fold change<1 and adjusted *p*-value<0.05). AV=Avicel® and SG=switchgrass.

|  | Total differentially regulated genes | Upregulated in co-culture | Downregulated in co-culture |
| --- | --- | --- | --- |
| *A. robustus* (AV) | 2937 | 1022 | 1915 |
| *A. robustus* (SG) | 237 | 135 | 102 |
| *C. churrovis* (SG) | 1151 | 579 | 333 |
