# Supplementary material for: Cocultivation of Anaerobic Fungi with Rumen Bacteria Establishes an Antagonistic Relationship
Source: mBio. 2021 Aug 17;12(4):e01442-21. doi: 10.1128/mBio.01442-21 (PMC8406330; doi:10.1128/mBio.01442-21)
Supplement: FIG S2 [file mbio.01442-21-sf002.docx]

**(A)**


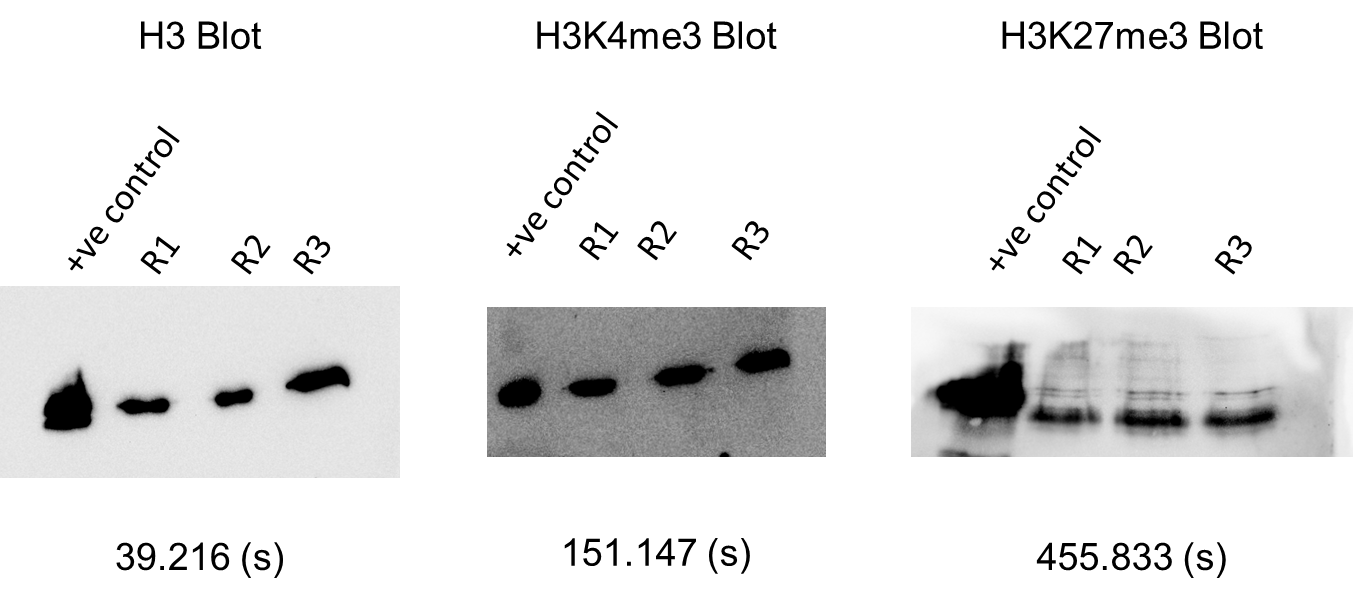


**(B)**

**
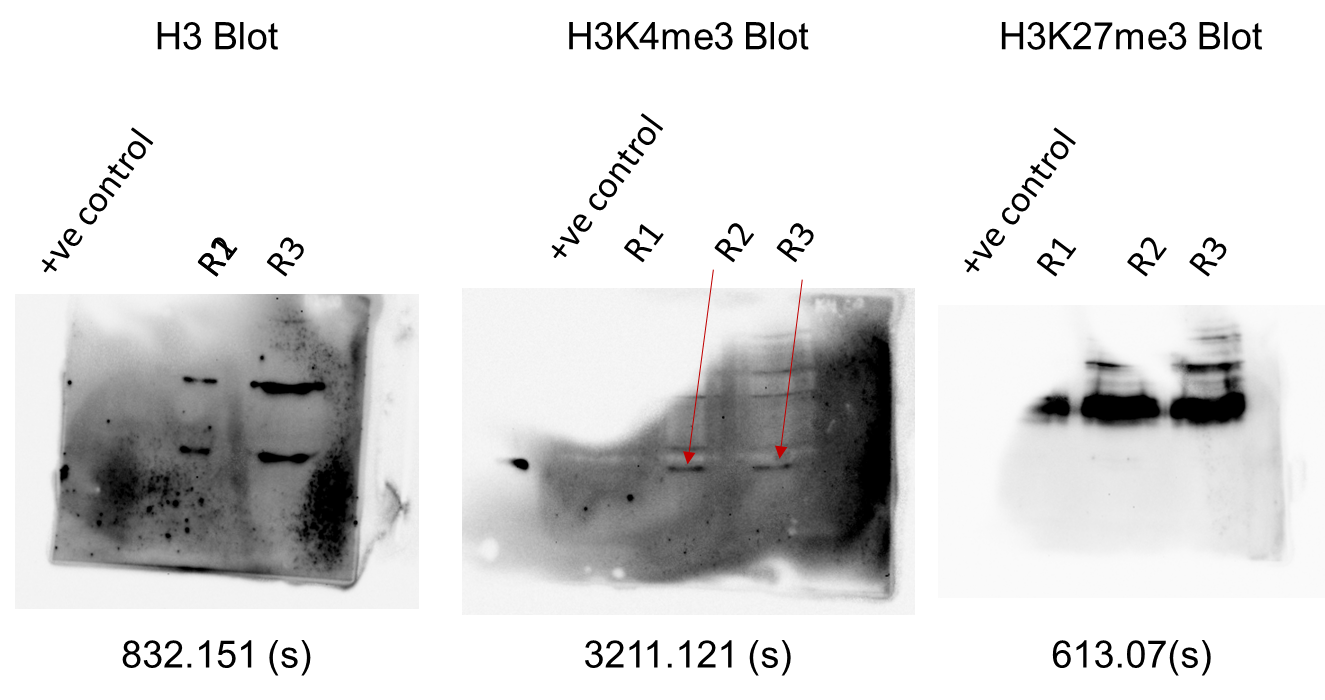
**

**Supplementary Figure S2. (A)** Western blot images of *A. robustus* monocultures grown on Avicel^®^ with rabbit antibodies against histone 3 (H3), trimethylated lysine 4 on histone 3 (H3K4me3), and trimethylated lysine 27 on histone 3 (H3K27me3). Exposure times are given below each blot. Lanes R1, R2, and R3 represent biological replicates of *A. robustus*. “+ve” indicates positive control: (left blot) rabbit anti-histone H3 antibody against *Candida glabarata*, (middle blot) rabbit anti-H3K4me3 against *Candida glabarata*, (right blot) rabbit anti-H3K27me3 against *Piromyces* sp. UH3-1. **(B)** Western blot images of *A. robustus* co-culture with *F.* sp. UWB7 grown on Avicel^®^ with rabbit antibodies against histone 3 (H3), trimethylated lysine 4 on histone 3 (H3K4me3), and trimethylated lysine 27 on histone 3 (H3K27me3). Exposure times are given below each blot. Lanes R1, R2, and R3 represent biological replicates of *A. robustus*. “+ve” indicates positive control: (left blot) rabbit anti-histone H3 antibody against *Candida glabarata*, (middle blot) rabbit anti-H3K4me3 against *Candida glabarata*, (right blot) rabbit anti-H3K27me3 against *Piromyces* sp. UH3-1. Replicates R2 and R3 are marked with red arrows for clarity in the middle blot.
