# Supplementary material for: Cocultivation of Anaerobic Fungi with Rumen Bacteria Establishes an Antagonistic Relationship
Source: mBio. 2021 Aug 17;12(4):e01442-21. doi: 10.1128/mBio.01442-21 (PMC8406330; doi:10.1128/mBio.01442-21)
Supplement: TABLE S4 [file mbio.01442-21-st004.docx]

**Supplementary Table S4.** Upregulated genes in *A. robustus* (co-culture with *F.* sp. UWB7 relative to *A. robustus* monoculture) assigned to the KOG class “Secondary metabolites biosynthesis, transport and catabolism” of. All cultures were grown with Avicel® as the substrate. Only genes with log_2_fold change greater than one and adjusted *p*-value less than 0.05 are shown. Genes on the same scaffold (bold) are co-localized, immediate neighbors. The multidrug/pheromone exporter class is KOG0055 and the nonribosomal peptide synthetase KOG is KOG1178.

| MycoCosm proteinId | Scaffold | log2FC | KOG name |
| --- | --- | --- | --- |
| 266148 | 47 | -7.1 | Multidrug/pheromone exporter, ABC superfamily |
| 275781 | 27 | -5.2 | Multidrug/pheromone exporter, ABC superfamily |
| 327152 | 110 | -3.7 | Multidrug/pheromone exporter, ABC superfamily |
| 330958 | 721 | -3.2 | Multidrug/pheromone exporter, ABC superfamily |
| 329547 | 322 | -3.1 | Multidrug/pheromone exporter, ABC superfamily |
| 305700 | **422** | -3 | Multidrug/pheromone exporter, ABC superfamily |
| 297543 | **434** | -2.1 | Multidrug resistance-associated protein/mitoxantrone resistance protein, ABC superfamily |
| 330191 | **422** | -1.6 | Multidrug/pheromone exporter, ABC superfamily |
| 265866 | 40 | -1.6 | Multidrug/pheromone exporter, ABC superfamily |
| 330237 | **434** | -1.4 | Multidrug resistance-associated protein/mitoxantrone resistance protein, ABC superfamily |
| 330852 | **626** | -1.4 | Multidrug resistance-associated protein/mitoxantrone resistance protein, ABC superfamily |
| 226999 | **626** | -0.96 | Multidrug resistance-associated protein/mitoxantrone resistance protein, ABC superfamily |
| 193122 | 480 | -2.9 | Non-ribosomal peptide synthetase/alpha-aminoadipate reductase and related enzymes |
| 294553 | 182 | -2.9 | Non-ribosomal peptide synthetase/alpha-aminoadipate reductase and related enzymes |
| 271076 | 279 | -2 | Non-ribosomal peptide synthetase/alpha-aminoadipate reductase and related enzymes |
| 231391 | **77** | -1.8 | Non-ribosomal peptide synthetase/alpha-aminoadipate reductase and related enzymes |
| 218823 | **77** | -1.2 | Non-ribosomal peptide synthetase/alpha-aminoadipate reductase and related enzymes |
| 330657 | 540 | -1.1 | Non-ribosomal peptide synthetase/alpha-aminoadipate reductase and related enzymes |
