# Supplementary material for: Cocultivation of Anaerobic Fungi with Rumen Bacteria Establishes an Antagonistic Relationship
Source: mBio. 2021 Aug 17;12(4):e01442-21. doi: 10.1128/mBio.01442-21 (PMC8406330; doi:10.1128/mBio.01442-21)
Supplement: FIG S3 [file mbio.01442-21-sf003.docx]

**(A)**


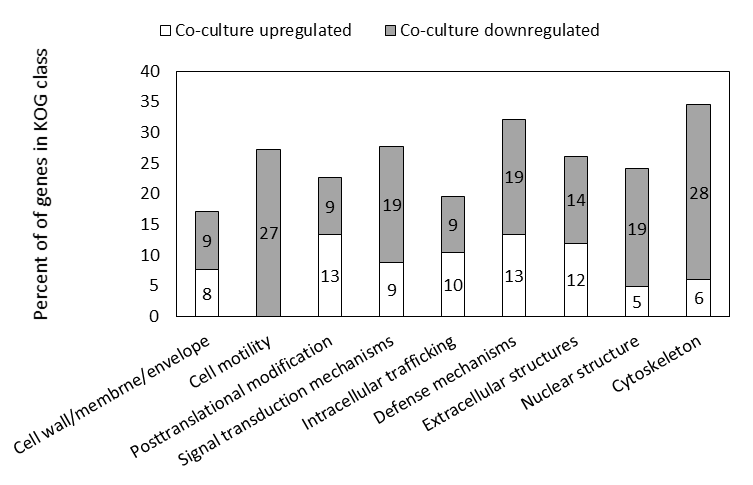


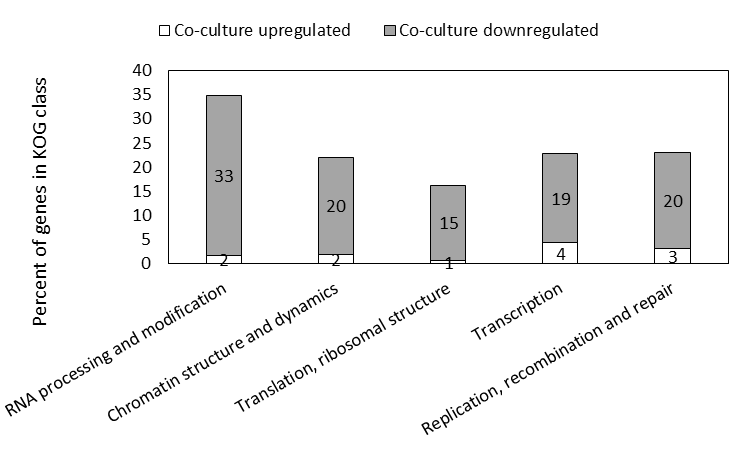


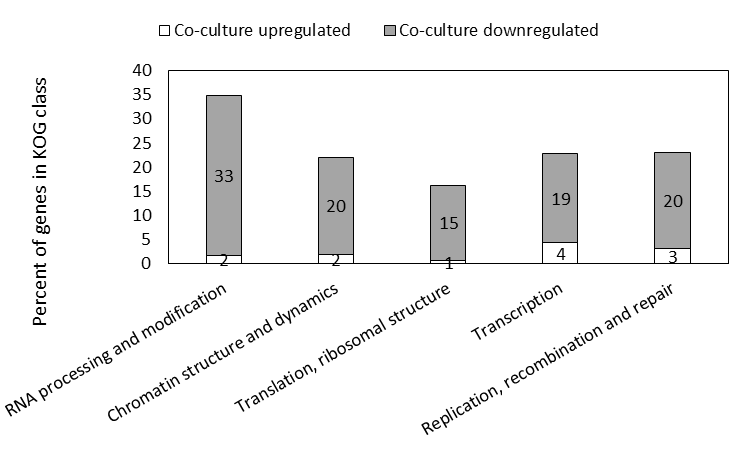


**(B)**

**
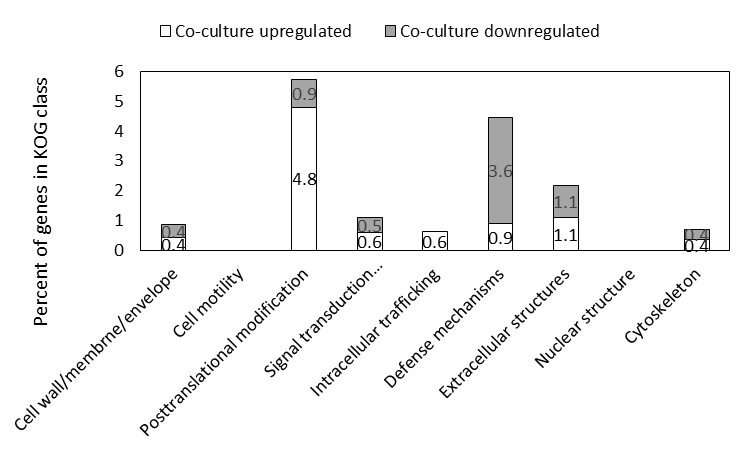
**

**
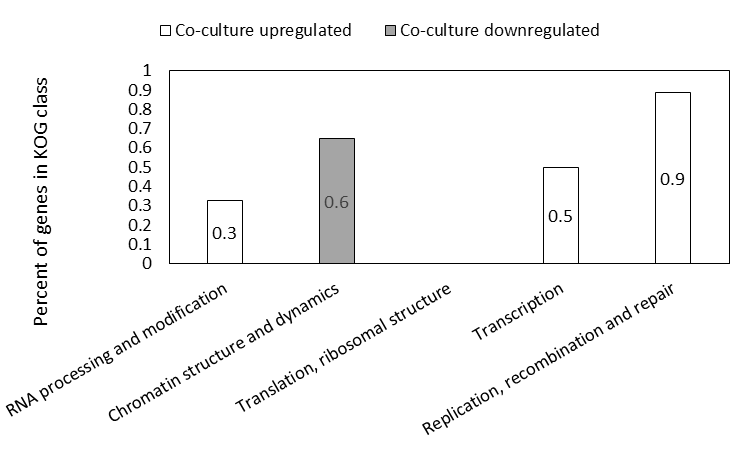
**

**
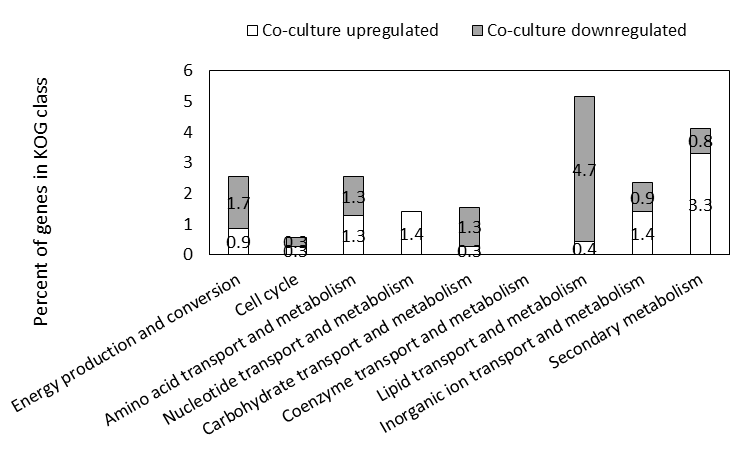
**

**(C)**

**
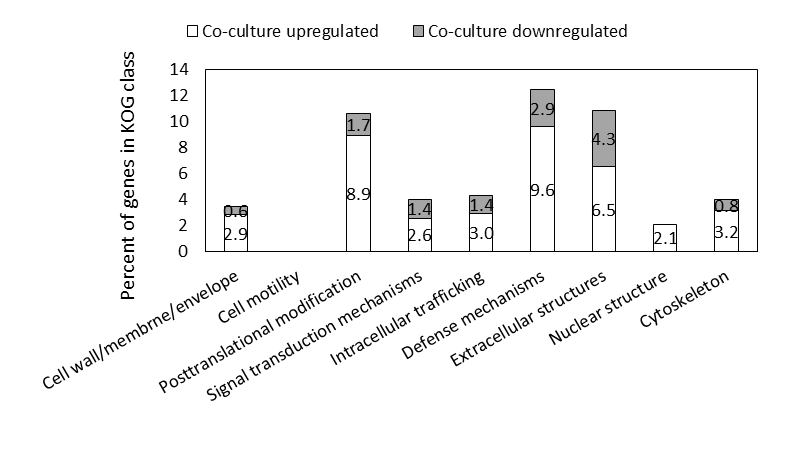
**

**
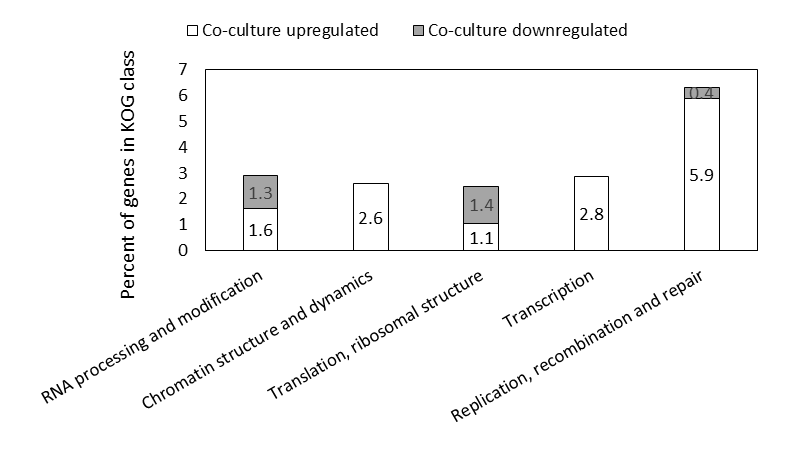
**

**
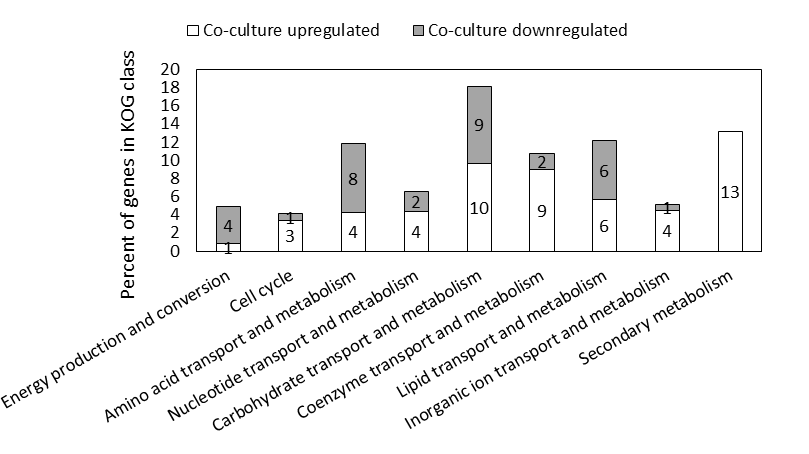
**

**Supplementary Figure S3.** Proportion of differentially expressed genes in each eukaryotic Orthologous Group (KOG) (1) class for anaerobic fungi in co-culture with *F.* sp. UWB7 relative to respective fungal monoculture. **(A)** *A. robustus* in co-culture with *F.* sp. UWB7 on Avicel^®^ relative to *A. robustus* monoculture on Avicel^®^ **(B)** *A. robustus* in co-culture with *F.* sp. UWB7 on switchgrass relative to *A. robustus* monoculture on switchgrass, **(C)** *C. churrovis* in co-culture with *F.* sp. UWB7 on switchgrass relative to *C. churrovis* monoculture on switchgrass. KOG classes are organized into three plots for each comparison: cellular processes and signaling (top), information storage and processing (middle), and metabolism (bottom). Bar labels signify the percent of genes upregulated (white) or downregulated (gray) in co-culture. All carbohydrate active enzymes (CAZymes) with catalytic domains were binned into the KOG class Carbohydrate transport and metabolism and all CAZymes without a catalytic domain were excluded from this analysis.

**References**

1. Koonin E, Fedorova N, Jackson J, Jacobs A, Krylov D, Makarova K, Mazumder R, Mekhedov S, Nikolskaya A, Rao B, Rogozin I, Smirnov S, Sorokin A, Sverdlov A, Vasudevan S, Wolf Y, Yin J, Natale D. 2004. A comprehensive evolutionary classification of proteins encoded in complete eukaryotic genomes. Genome Biol 5:R7.
