# Supplementary material for: Cocultivation of Anaerobic Fungi with Rumen Bacteria Establishes an Antagonistic Relationship
Source: mBio. 2021 Aug 17;12(4):e01442-21. doi: 10.1128/mBio.01442-21 (PMC8406330; doi:10.1128/mBio.01442-21)
Supplement: FIG S4 [file mbio.01442-21-sf004.docx]

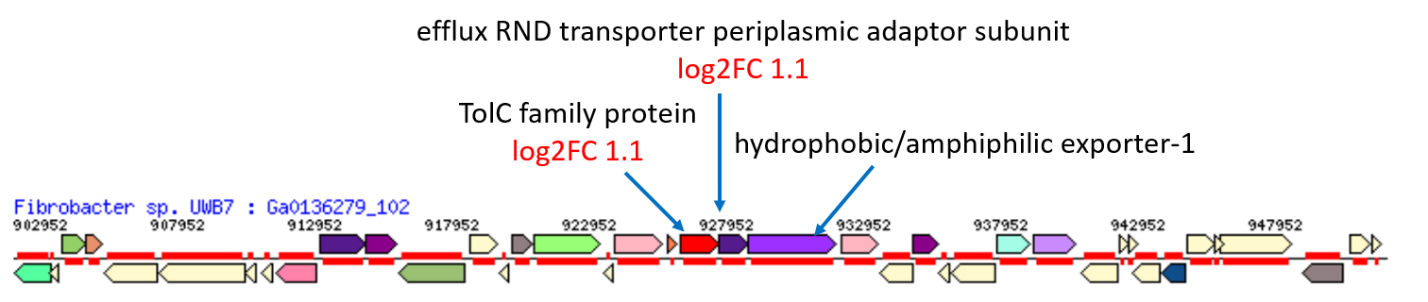


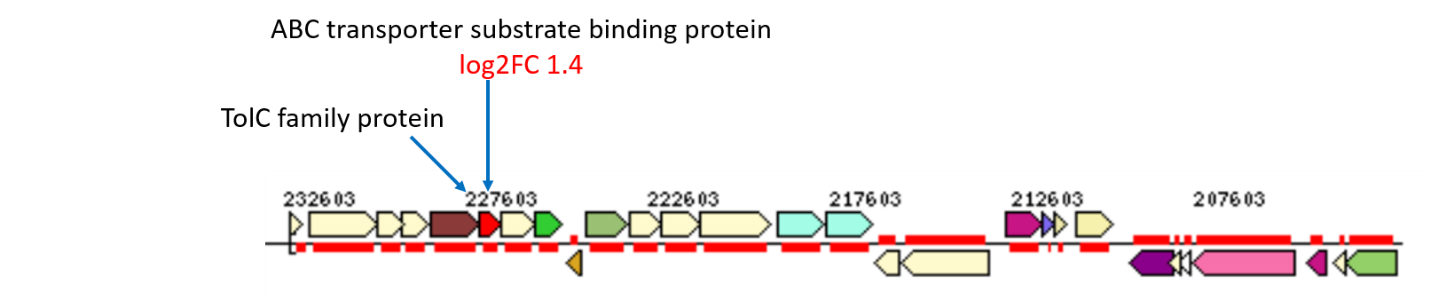


**Supplementary Figure S4.** Gene neighborhoods visualized using IMG/M(1) for Ga0136279_1902 (efflux RNA transporter periplasmic adapter subunit, log_2_ fold change 1.1 for co-culture vs. monoculture) and Ga0136279_2080 (ABC transporter substrate binding protein, log_2_fold change 1.4 for co-culture versus monoculture).

**References**

1. Chen IMA, Chu K, Palaniappan K, Pillay M, Ratner A, Huang J, Huntemann M, Varghese N, White JR, Seshadri R, Smirnova T, Kirton E, Jungbluth SP, Woyke T, Eloe-Fadrosh EA, Ivanova NN, Kyrpides NC. 2019. IMG/M v.5.0: An integrated data management and comparative analysis system for microbial genomes and microbiomes. Nucleic Acids Res 47:D666–D677.
