# Supplementary material for: Cocultivation of Anaerobic Fungi with Rumen Bacteria Establishes an Antagonistic Relationship
Source: mBio. 2021 Aug 17;12(4):e01442-21. doi: 10.1128/mBio.01442-21 (PMC8406330; doi:10.1128/mBio.01442-21)
Supplement: FIG S5 [file mbio.01442-21-sf005.docx]

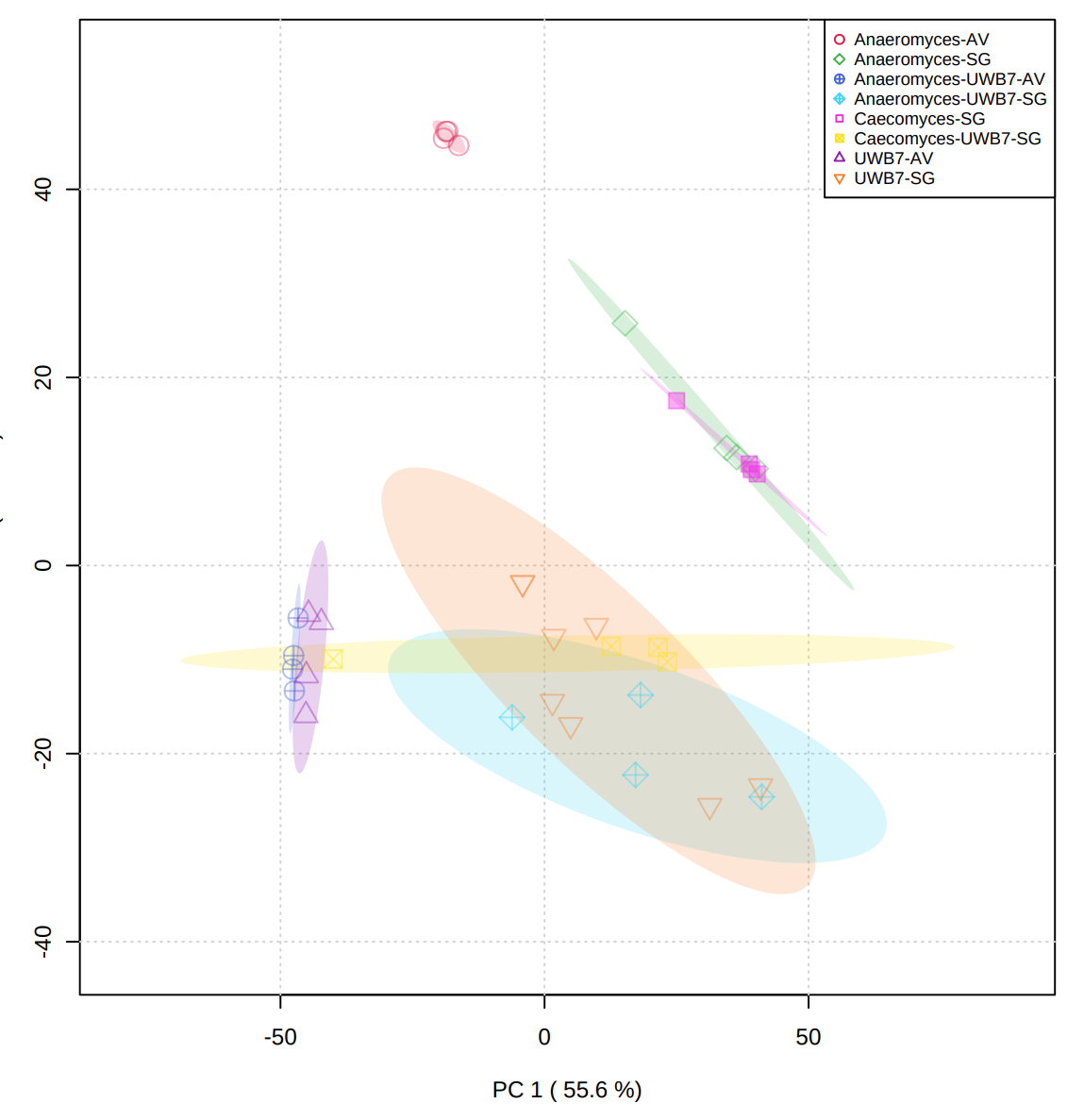


**Supplementary Figure S5.** Two-dimensional principal component analysis (PCA) plots of the untargeted nonpolar metabolomics data obtained for co-cultures and monocultures of *A. robustus*, *C. churrovis*, and *F*. sp. UWB7. UWB7=*F*. sp. UWB7, AV=Avicel^®^, SG=switchgrass. Empty symbols indicate monocultures, whereas symbols with interior crosses indicate co-cultures. Ellipses represent 95% confidence regions. Plots were rendered by MetaboAnalyst (1).

**References**

1. Xia J, Sinelnikov I V, Han B, Wishart DS. 2015. MetaboAnalyst 3 . 0 –– making metabolomics more meaningful. Nucleic Acids Res 43:251–257.
