# Supplementary material for: Cocultivation of Anaerobic Fungi with Rumen Bacteria Establishes an Antagonistic Relationship
Source: mBio. 2021 Aug 17;12(4):e01442-21. doi: 10.1128/mBio.01442-21 (PMC8406330; doi:10.1128/mBio.01442-21)
Supplement: TEXT S1 [file mbio.01442-21-s0001.docx]

**Supplementary Text**

*Evaluation of polyA selection and ribosomal depletion effectiveness*

The dual ribosomal depletion method described in the main text methods was highly effective in the co-cultures of *C. churrovis* with *F.* sp. UWB7, resulting in only 15-20% rRNA reads. In order to compensate for the presence of some rRNA reads, co-cultures were sequenced at a greater depth than monocultures. Differential expression analysis by DESeq2 accounts for these differences in sequencing depth (1). Dual ribosomal depletion was poor in co-cultures of *A. robustus* and *F.* sp. UWB7, resulting in ~60% ribosomal reads. Therefore, no differential expression analysis was conducted for *F.* sp. UWB7 genes in co-cultures with *A. robustus* compared to monocultures. All polyA-selected libraries had less than 2% ribosomal RNA.

*Transcriptomic analysis of fungal and bacterial carbohydrate active enzymes in co-culture compared to monoculture*

In response to co-cultivation with *F.* sp. UWB7 on Avicel^®^, *A. robustus* upregulated 85 carbohydrate active enzymes (CAZymes) and downregulated 105 CAZymes (~10% and 12% of total predicted CAZymes), with log_2_ fold change >1 and adjusted *p*-value <0.05. Upregulated CAZymes included 18 predicted xylanases from glycoside hydrolase (GH) families 11, 39, and 43 (<http://cazy.org/>). Despite the upregulation of xylanases, it cannot be ruled out that the upregulation of CAZymes in co-culture is due to the carryover Avicel^®^ particles during inoculation with *F.* sp. UWB7, since it has been previously shown that *A. robustus* co-regulates hemicellulases and cellulases even during growth on cellobiose (5).  Similarly, glucose carryover from *F.* sp. UWB7 inoculum may cause CAZyme repression, which has been demonstrated previously for anaerobic gut fungi (6). By the same thresholds for statistical significance, less than ten CAZymes were upregulated in co-culture either for *A. robustus* or *C. churrovis* grown on switchgrass, which suggesting that a stronger response is elicited from the fungus when *F.* sp. UWB7 is grown on the preferred substrate of Avicel^®^ in co-culture.

In contrast, *F.* sp. UWB7 downregulates genes encoding enzymes proposed to be involved in biomass deconstruction. A total of 17 CAZymes were downregulated in co-culture with *C. churrovis*, in addition to one predicted protein containing a fibro-slime domain, which is thought to play a role in adhesion to cellulose (7). We hypothesize that the presence of excess sugars released from the plant biomass by the fungi repress CAZymes of *F. sp. UWB7*. The CAZymes of anaerobic gut fungi are similarly catabolite repressed (6), and previous work has also shown that anaerobic gut fungi release sufficient sugars from plant biomass to support the growth of other microorganisms such as *Saccharomyc* *es cerevisiae* (5).

**References**

1. Love MI, Huber W, Anders S. 2014. Moderated estimation of fold change and dispersion for RNA-seq data with DESeq2. Genome Biol 15:550.

2. Kopylova E, Noe L. 2012. SortMeRNA : fast and accurate filtering of ribosomal RNAs in metatranscriptomic data 28:3211–3217.

3. Quast C, Pruesse E, Yilmaz P, Gerken J, Schweer T, Yarza P, Peplies J, Glöckner FO. 2013. The SILVA ribosomal RNA gene database project: Improved data processing and web-based tools. Nucleic Acids Res 41:D590–D596.

4. Griffiths-Jones S, Bateman A, Marshall M, Khanna A, Eddy SR. 2003. Rfam: An RNA family database. Nucleic Acids Res. Oxford Academic.

5. Henske JK, Wilken SE, Solomon K V., Smallwood CR, Shutthanandan V, Evans JE, Theodorou MK, O’Malley MA. 2018. Metabolic characterization of anaerobic fungi provides a path forward for bioprocessing of crude lignocellulose. Biotechnol Bioeng 115:874–884.

6. Henske JK, Gilmore SP, Haitjema CH, Solomon K V, Malley MAO. 2018. Biomass-Degrading Enzymes Are Catabolite Repressed in Anaerobic Gut Fungi. AIChE J 64.

7. Burnet MC, Dohnalkova AC, Neumann AP, Lipton MS, Smith RD, Suen G, Callister SJ. 2015. Evaluating models of cellulose degradation by Fibrobacter succinogenes S85. PLoS One 10:1–19.
